# Sex differences in familial risk and genetic components of suicide attempts: a register-based cohort study in Sweden

**DOI:** 10.1136/bmjment-2025-302082

**Published:** 2026-03-10

**Authors:** Thuy-Dung Nguyen, Tong Gong, Kejia Hu, Ralf Kuja-Halkola, Karen Borges, Agnieszka Butwicka, Isabell Brikell, James J Crowley, Zheng Chang, Brian M D’Onofrio, Henrik Larsson, Paul Lichtenstein, Christian Rück, Cynthia Bulik, Fang Fang, Patrick Sullivan, Yi Lu

**Affiliations:** 1 Department of Medical Epidemiology and Biostatistics, Karolinska Institutet, Stockholm, Sweden; 2 Institute of Environmental Medicine, Karolinska Institutet, Stockholm, Sweden; 3 Sleep Medicine Center, Mental Health Center, Sleep Medicine Laboratory, West China Hospital, Sichuan University, Chengdu, Sichuan, China; 4 West China Biomedical Big Data Center, West China Hospital, Sichuan University, Chengdu, Sichuan, China; 5 Division of Psychiatry, University of Edinburg, Edinburgh, UK; 6 Department of Psychiatry, Mayo Clinic, Rochester, New York, USA; 7 Division of Mental Health Services, Akershus University Hospital, Lørenskog, Norway; 8 Institute of Clinical Medicine, University of Oslo, Oslo, Norway; 9 Department of Biostatistics and Translational Medicine, Medical University of Lodz, Lodz, Poland; 10 Global Public Health and Primary Care, University of Bergen, Bergen, Norway; 11 Department of Biomedicine, Aarhus University, Aarhus, Denmark; 12 Department of Genetics, University of North Carolina at Chapel Hill, Chapel Hill, North Carolina, USA; 13 Department of Psychiatry, University of North Carolina at Chapel Hill, Chapel Hill, North Carolina, USA; 14 Department of Clinical Neuroscience, Center for Psychiatry Research, Karolinska Institutet, Stockholm, Sweden; 15 Department of Psychological, Indiana University Bloomington, Bloomington, Indiana, USA; 16 School of Medical Sciences, Örebro universitet, Örebro, Sweden; 17 Stockholm Health Care Services, Region Stockholm, Stockholm, Sweden; 18 Department of Nutrition, University of North Carolina at Chapel Hill, Chapel Hill, North Carolina, USA

**Keywords:** Psychiatry, Mental Health

## Abstract

**Background:**

Suicidal behaviour shows notable sex differences, and understanding whether genetic factors contribute to these differences is critical for identifying at-risk individuals and prevention.

**Objective:**

We aim to investigate the genetic contribution to suicide attempts and examine whether genetics account for sex differences in incidence.

**Methods:**

This population-based cohort study includes 3.1 million individuals born 1963–1998 and followed through Swedish National Registers, including hospitals and specialist outpatient diagnoses and cause of death data. Suicide attempts were identified using ICD codes, indicating intentional self-harm, self-harm using lethal methods or leading to hospitalisation, or resulting in death. Familial aggregation, coaggregation, pedigree heritability and genetic correlations were estimated using genealogical data. For sex-specific analyses, we examined mother–daughter, female sibling, father–son and male sibling pairs, separately.

**Findings:**

Suicide attempts were more common among females than males (3.3% vs 2.6%). In both sexes, risk aggregated within families (ORs ranged 1.6–3.4 across relative types) and was higher in first-degree than second-degree relatives. Familial aggregation was stronger in females than in males, and in same-sex first degree relatives compared with cross-sex pairs. Pedigree heritability was 41.9% (95% CI 36.0 to 48.4%) and did not differ significantly by sex (female 51.4% (95% CI 40.1% to 58.6%), male 45.1% (95% CI 32.3% to 52.5%), Bootstrap p value 0.40). Suicide attempt showed moderate to high pedigree genetic correlations with psychiatric disorders, strongest with substance use disorders (SUD, r_g_=0.85 (95% CI 0.83 to 0.96)), with no significant sex differences. The genetic correlation between female and male suicide attempts was high (0.85 (95% CI 0.80 to 0.99)), suggesting a substantial genetic overlap.

**Conclusions:**

Suicide attempt has a moderate heritable component that largely overlaps between females and males and with other psychiatric disorders, particularly SUD. Stronger familial aggregation in females and in same-sex pairs highlights the potential role of sex-specific environmental or social factors. Future research should focus on non-genetic contributors and their potential interaction with genetic factors to better understand and address sex disparities in suicidal behaviour

**Clinical implications:**

Genetic risk for suicide attempt is substantial but does not fully explain sex differences in incidence. Clinicians should, therefore, consider non-genetic, including sex-specific environmental and social factors, alongside family history and psychiatric comorbidity when assessing suicidal risk.

**WHAT IS ALREADY KNOWN ON THIS TOPIC**

- Suicide is a major global public health issue—one of the leading causes of death and a source of deep psychological impact on families and communities.
- Suicidal behaviours show clear sex differences: males die by suicide more often, while females attempt suicide at about two times the rate of males.
- Environmental risk and protective factors—such as psychiatric illness, trauma and socioeconomic status—only partly account for these sex disparities in suicide attempts.
- Given that suicidal behaviour is both familial and heritable, investigating genetic factors may clarify sex differences and guide more personalised prevention strategies considering, eg, sex-specific risk factors, familial and genetic risks.

**WHAT THIS STUDY ADDS**

- This study is among the few to comprehensively investigate sex differences in the genetics of suicide attempt, using nationwide longitudinal register data and classical family-based designs.
- We observed stronger familial aggregation among females and same-sex first-degree relatives, even though overall heritability of suicide attempts did not differ by sex, suggesting potential sex-specific effects of shared familial environment and possibly other social factors. These findings have strong clinical implications: the sex of affected relatives should be taken into account when assessing suicide risk.
- While genetic factors significantly influence suicide attempt risk, they do not fully account for observed sex differences—underscoring the need to investigate non-genetic factors and gene–environment interactions.

**HOW THIS STUDY MIGHT AFFECT RESEARCH, PRACTICE OR POLICY**

- Suicide prevention efforts and clinical risk assessment should acknowledge not only the moderate genetic risk but also the sex-specific environmental or social factors contributing to suicide attempts.
- It underscores the need for research and clinical practice to integrate genetic vulnerability with social and environmental determinants, such as family dynamics and exposure to trauma, to better understand and address sex-specific risk factors in suicidal behaviour.

## Background

 Suicide is a critical public health concern, with an age-standardised rate at 9 per 100 000 population, accounting for approximately 700 000 deaths globally each year.^1^ Suicide attempts are even more common, with an estimated lifetime prevalence of 2.7%–3.3% across national samples.^2^ Notably, a previous history of suicide attempts is one of the strongest predictors of subsequent death by suicide.^3^ Therefore, improving understanding of determinants of suicide attempts is central to population-level suicide prevention and monitoring. This is directly relevant to global health frameworks, including the UN Sustainable Development Goals, where suicide rate is used to monitor the progress on promoting mental health and well-being.

Suicidal behaviours differ between males and females. Males account for most suicide deaths,^4^ whereas the prevalence of suicide attempts is two times higher among females than males.^5^ In a previous study, also conducted using the Swedish registers,^6^ we observed sex differences in the lifetime prevalence of suicide attempts, with females showing a higher cumulative incidence (4.91% vs 4.27% in males) and a higher incidence rate before age 24. Like the comprehensive biopsychosocial model of suicide risk,^7^ the factors contributing to sex differences in suicidal behaviour are likely multifaceted. For example, neurobiological alterations, such as dysregulations of glutamatergic system, have been reported to be more pronounced in female patients with major depressive disorder who died by suicide.^8^ Recent review, including experimental studies in adults and correlational studies in adolescents, also implicates puberty-related individual differences in neurobiological sensitivity to reproductive hormonal fluctuations as potential contributors to sex-specific development of brain circuits involved in emotion regulation and impulse control, which may in turn influence suicidal behaviour.^9^ Additionally, previous research synthesised from international studies has shown that differences in environmental risk and protective factors—such as socioeconomic status, psychiatric diagnoses, trauma, family history or differential social norms—only partially explain the sex difference in the incidence of suicide attempts.^10^ Cross-national work based on global ecological data from 182 countries indicates that sex-specific cultural expectations, help seeking, access to and choice of methods and reporting practices can also shape these gaps,^11^ highlighting the value of international evidence when interpreting sex differences and planning prevention.

Heritability estimates for suicide attempts from twin studies vary, ranging from 17% to 55% (online supplemental Table S1). While prior research conducted in Sweden has identified sex differences in the genetics of major suicide risk factors such as depression and substance use,^12^ it remains unclear whether the observed sex differences in the incidence of suicide attempt reflect underlying genetic influences. Evidence to date is mixed. One study, also conducted in Sweden, reported higher risk of suicide attempts among male than female offspring of parents who had attempted suicide,^13^ and another reported a stronger genetic correlation between suicide attempts and suicide deaths in males (*r_g_*=0.74; 95% CI 0.63 to 0.87) than in females (*r_g_*=0.67; 95% CI 0.55 to 0.67).^5^ In contrast, a large Swedish register study found only modest sex differences in heritability, with overlapping CIs 52% in females (95% CI 44% to 56%) vs. 41% in males (95% CI=38–49%).^5^ These mixed findings underscore the need to clarify the role of sex and relative type in the familial aggregation of suicide attempts and their potential genetic underpinnings.

## Objectives

In this study, we used Swedish population-based registers to extract a birth cohort of over three million individuals (born 1963–1998) and their biological relatives. Leveraging comprehensive longitudinal health records, we aimed to (1) investigate the genetic basis of suicide attempts and (2) assess the role of genetics in explaining sex differences in the incidence of attempting suicide, using measures of familial aggregation, coaggregation, pedigree heritability and genetic correlations. We primarily focused on a strict definition of suicide attempt (N=89 278; 2.9% of the birth cohort), capturing severe cases characterised by high-lethality methods, and intensive medical treatment, or resulting in death.^14^,^17^ For comparison, we also examined a broader definition of self-harm, encompassing both non-lethal or unclear-intent events commonly included in the existing literature.

## Methods

### Data

This study used data from Swedish national registers (online supplemental Table S2), updated until 31 December 2019. Personal identity numbers, assigned at birth or immigration, were used to link individuals across registries. Records of suicide attempt and psychiatric disorders were identified from the National Patient Register (NPR) using ICD codes. The NPR captures inpatient psychiatric care since 1973 and outpatient care since 2001. Records of deaths by suicide and other causes were obtained from the Cause of Death Register (CDR), which covers all deaths in Sweden since 1952. We identified family relationships using the Multi-Generation Register and birth data using the Swedish Medical Birth Register. All registers were linked to the Total Population Register, which tracks migration for all residents.

### Study population

The index population included individuals born in Sweden between 1 January 1963 and 31 December 1998, who were alive and had not emigrated by age 10 (online supplemental Figure S1). Individuals were followed from age 10 until 31 December 2019. The chosen birth cohort ensures that individuals were at least 10-year old when the NPR began systematically capturing inpatient psychiatric care in 1973, and at least 21 years old by the end of the follow-up. We chose the lower age limit at 10, because suicide before age 10 is rare in Sweden, and accidental ingestions by children (98% occur before age 10) are classified as ‘self-harm of undetermined intent’. The cohort was selected to (1) capture the peak age of suicide attempt (15–24 years), (2) identify sufficient suicide attempt cases and (3) ensure enough follow-up to avoid missing cases. While immigrants are an important population for suicide research,^18^ we included only individuals born in Sweden as the main study population for practical considerations, for example, to minimise the impact of missing parental information or data censoring related to immigration.

All parents, full and half siblings of the index individuals were included to maximise sample size. Relatives born outside the chosen cohort were included for familial aggregation and coaggregation analyses, but those who died, migrated or had not reached age 10 by 31 December 2019 were excluded. For heritability and genetic correlation estimates, only sibling pairs born during 1963–1998 were included. Family clusters were included in the analyses to avoid underestimation of the CIs. Families were defined by linking parents through spouses and siblings through at least one parent, resulting in large, mutually exclusive families within the parent–offspring generation.

### Phenotype definitions

Suicide attempts were defined using data from the Swedish NPR and CDR, building on prior Swedish research^19^ with modifications to identify medically serious cases. As detailed in our previous work,^6^ persons with suicide attempt included individuals meeting specific criteria based on patient and death records. Specifically, suicide attempt comprised individuals with: (1) Patient Register records of intentional self-poisoning or self-harm (ICD codes X60-X84); (2) Patient Register records of self-harm with undetermined intent (ICD codes Y10-Y34), events with unclear intent (ICD codes E980-E989), sequelae of self-harm (ICD codes 87.0, Y87.2), suicide attempts and self-inflicted injuries (ICD codes E950-E959) *with* a lethal method of self-harm *and/or* resulted in inpatient care or (3) death by self-harm or suicidal behaviour (ICD codes X, Y, E) (online supplemental Table S3).

Additionally, to facilitate comparison with other studies,^19^ a broader phenotype of self-harm, including suicide attempts, was also defined using relevant ICD codes from NPR and CDR (same ICD codes as above, but not restricting on the lethal method or hospitalisation). Psychiatric disorders were also identified from the Patient Register using ICD codes (online supplemental Table S3).

### Statistical methods

A quick overview of the statistical methods is summarised in online supplemental Table S4.

#### Familial aggregation and coaggregation

For suicide attempt, we estimated its familial aggregation and coaggregation with 11 psychiatric disorders that are key psychiatric risk factors for suicide,^19^ including substance use disorder (SUD), post-traumatic stress disorder (PTSD), major depressive disorder (MDD), anxiety, obsessive compulsive disorder (OCD), bipolar disorder, schizophrenia, other psychotic disorders (excluding schizophrenia), attention-deficit hyperactivity disorder (ADHD), autistic spectrum disorder and eating disorders. For families with multiple sibling pairs, we kept all possible pairs for the aggregation and coaggregation analyses. We used generalised estimating equations with a logit link function to estimate ORs and robust SEs were estimated by clustering families using the R package *drgee*.

We examined five types of familial relationships: mother–offspring, father–offspring, full siblings, maternal half-siblings and paternal half-siblings. For each relationship type, pairs were used two times if both were born during 1963–1998, switching roles as index and relative, and once if the relative was born outside this period. In the analyses, suicide attempt in the index individual was the outcome variable, regressed on suicide attempt or psychiatric disorders in their relatives. For sex-specific analyses, we separated mother–daughter and female sibling pairs, and father–son and male sibling pairs. Models including both sexes adjusted for sex and birth year, while sex-specific models adjusted only for birth year of the index individual and their relative.

#### Heritability and genetic correlation

We applied structural equation models (SEM) to estimate the pedigree heritability (*h*^2^) of suicide attempt using the liability threshold model and genetic correlations (*r_g_*) between suicide attempt and psychiatric disorders, comparing full-siblings and maternal half-siblings. The model relied on the assumption of no interaction between genetic and environmental effects. For families with multiple sibling pairs, we randomly selected one pair of siblings to fit the SEM. We first fit models with three variance components: additive genetic (A), shared environment (C) and unique environment (E, including measurement error), commonly referred to as ACE models. Full siblings were assumed to share 50% of the additive genetic component, while maternal half-siblings shared 25%. Both sets of siblings were assumed to share 100% of their common environment as, in Sweden, the majority of full-siblings and maternal half-siblings grew up in the same household (while paternal half-siblings were less likely to be reared together).^20^ To account for dependency within families, we used bootstrap resampling (1000 replicates) with families as the sampling unit, separately for full and maternal half-siblings. 95% CIs, potentially asymmetrical, were defined by the 26th to 975th sorted bootstrap estimates.

For heritability estimation, both ACE and AE univariate models were fitted using weighted least squares (WLS) estimation for Linear Structural Relations (LISREL) models in the OpenMx software V.2.19.8. Models for both sexes adjusted for sex and birth year, while sex-specific models adjusted for birth year only. We compared model fit between ACE and AE using the χ^2^ test.

For genetic correlations estimation, we fitted two types of bivariate models. For the *r_g_* between suicide attempt and psychiatric disorders, we applied the standard bivariate WLS models, adjusting for sex and birth year, and compared model fit between ACE and AE using χ^2^ test. As these analyses were intended to characterise the broad and sex-specific pattern of shared genetic liability between suicide attempt and psychiatric disorders rather than to test specific a priori hypotheses, we focused on the relative magnitude of the *r_g_* and did not apply multiple-testing correction. For the genetic correlation between male and female suicide attempt, we built a special type of model using maximum likelihood; details about model setup have been published elsewhere.^21^ All models accounted for birth year and we compared model fit using likelihood ratio test. For consistency, estimates from ACE models were presented in the main text for heritability and genetic correlations.

### Sensitivity analysis

Consider the sex difference in age at initial suicide attempt which was found in our prior study,^6^ we presented results of familial aggregation further stratified by relative’s age at first attempt (10–18, 19–25, >25 year) and by sex.

To evaluate whether findings are generalisable to immigrant families, we broadened the index population to include immigrants born 1963–1998 and migrated to Sweden before age 10, and their foreign-born parents and siblings who also migrated before age 10. As a demonstration, we report results on familial aggregation.

To account for the timing of suicide attempts within families and to properly handle censoring, we conducted a sensitivity analysis treating suicide attempt as a time-to-event outcome. Using Cox proportional hazards models with age of the index person as the time scale, we estimated HRs where censor for follow-up was made at emigration, death or end of the study. Robust SEs were used to account for family clustering.

### Patient and public involvement

This study used precollected data from national registers; therefore, no patients were involved in formulating the research question, selecting outcome measures or contributing to the design, conduct or interpretation of the study. The findings will be communicated to the Swedish public through media outreach (eg, press releases and other communications) on publication.

## Findings

We present results for suicide attempt in the main text, and the majority of results for any self-harm including suicide attempts in the *Supplement*. We defined a birth cohort of 3 058 374 Sweden-born individuals as the index population (online supplemental Figure S1). The cohort contained 48.6% females and age at the end of follow-up ranged from 21 to 57 years (mean 39.6, SD 10.5, median 39.8). In this cohort, 89 278 (2.9% of population) individuals had at least one suicide attempt (55.0% of cases were female); and 126 411 (4.1% of population) individuals had self-harm (50.0% of cases were female) (online supplemental Table S5).

Females and males had similar age at end of follow-up. Females had a higher proportion of suicide attempts compared with males (F: 3.3% vs M: 2.6%). In contrast, the difference in proportion of self-harm between the two sexes was minimal (F: 4.2% vs M: 4.1%) (online supplemental Table S5).

Psychiatric disorders were more common among individuals with suicide attempt (75.6% had at least one diagnosis) compared with those without (15.2%). Among those with suicide attempt, the frequency of specific psychiatric disorders ranged from 3.9% (schizophrenia) to 46.3% (MDD) and 46.5% (SUD). Psychiatric disorders were more common among females who attempted suicide than males (79.0% vs 71.5% with at least one diagnosis), with higher prevalence of MDD, PTSD, anxiety, bipolar disorder, eating disorders and OCD (online supplemental Table S6).

### Familial aggregation and coaggregation

**Figure 1:**
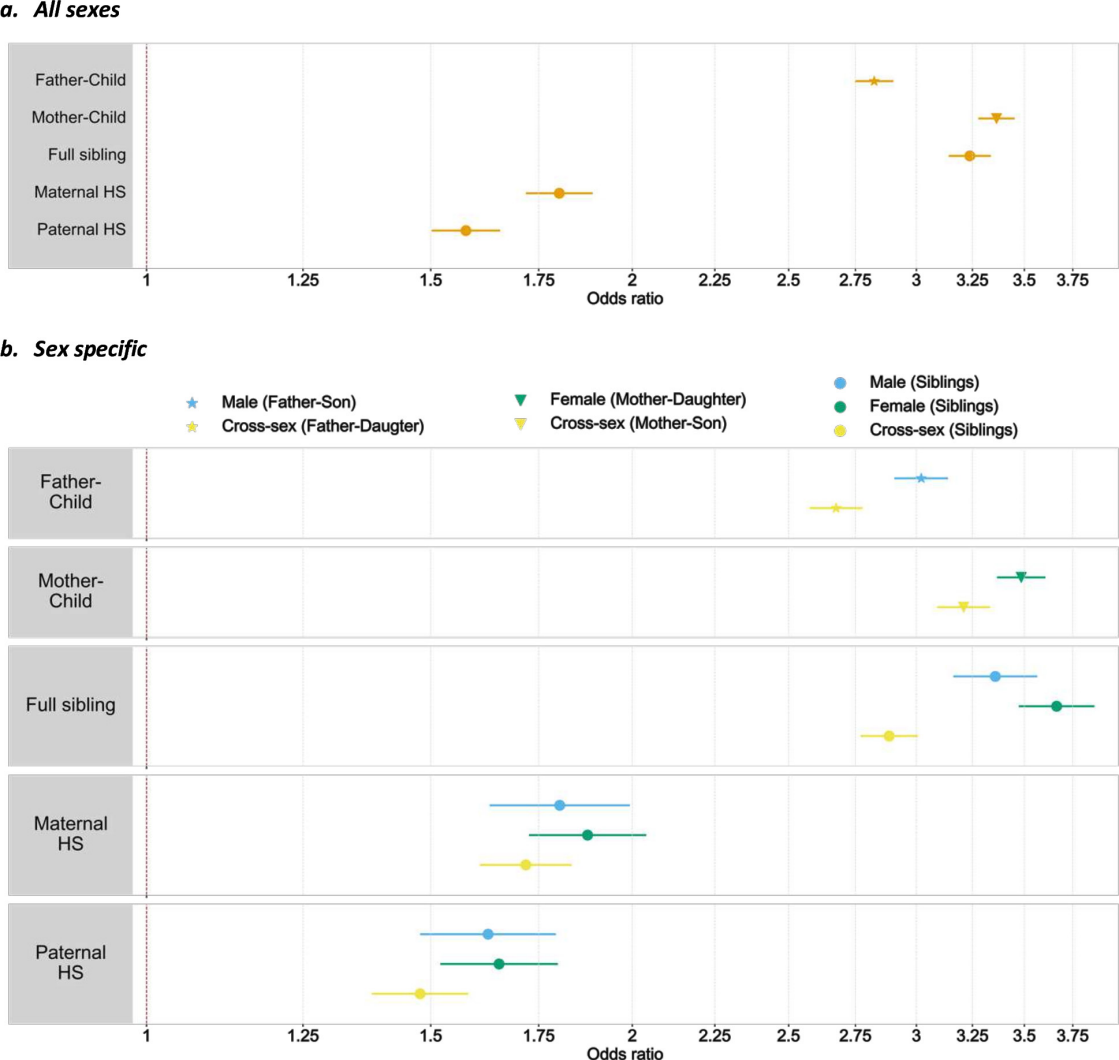
Familial aggregation of suicide attempt for all sexes (panel a) and for sex specific (panel b). Markers show point estimates, error bars show 95% CI for the estimates. Markers of different shapes represent types of relative. Colours represent sex groups. HS, half-siblings.

We identified a total of 3 653 013 mother–offspring pairs, 3 477 548 father–offspring pairs, 4 992 249 full-sibling pairs, 908 740 maternal half-sibling pairs and 1 164 125 paternal half-sibling pairs for familial risk analyses. Suicide attempt risk was elevated among relatives of affected individuals, highest in mother–offspring pairs (OR = 3.36, 95% CI 3.28 to 3.45). First-degree relatives (parents–offspring, full siblings) showed higher ORs than second-degree relatives (half-siblings), indicating a role for genetic factors. Risk was greater among maternal (OR=1.80, 95% CI 1.72 to 1.89) than paternal half-siblings (OR=1.58, 95% CI 1.50 to 1.66), consistent with greater shared environment as in Sweden, maternal-half-siblings are more likely to live together than paternal half-siblings^20^ (figure 1, panel a; online supplemental Table S7a).

Among first-degree relatives, there were different patterns of familial aggregation between sexes (table 1). The highest OR was observed in female full-sibling pairs (OR 3.7, 95% CI 3.5 to 3.9). ORs were generally higher in female relative pairs (mother–daughter, female siblings) than male pairs (father–son, male siblings) (figure 1, panel b; table 1).

**Table 1:** Sex-specific familial risk of suicide attempt

Relative types	Number of pairs analysed	N event	Proportion of events: unexposed	Proportion of events: exposed	OR (95% CI)*
Males					
Father–son	1 787 126	47 508	2.51%	7.18%	3.02 (2.91 to 3.14)
Male full sibling	1 320 878	33 905	2.42%	8.02%	3.36 (3.16 to 3.57)
Male maternal half-sibling	237 961	12 160	4.94%	8.62%	1.80 (1.63 to 1.99)
Male paternal half-sibling	303 544	13 870	4.46%	7.14%	1.63 (1.48 to 1.79)
Females					
Mother–daughter	1 775 774	60 801	3.19%	10.38%	3.48 (3.37 to 3.61)
Female full sibling	1 175 910	39 532	3.12%	10.57%	3.66 (3.47 to 3.87)
Female maternal half-sibling	216 430	14 746	6.51%	11.41%	1.88 (1.73 to 2.04)
Female paternal half-sibling	279 280	16 548	5.76%	9.03%	1.65 (1.52 to 1.80)
Cross-sex					
Mother-son	1 877 239	52 827	2.65%	7.83%	3.21 (3.09 to 3.33)
Father–daughter	1 690 422	55 823	3.14%	7.99%	2.68 (2.58 to 2.78)
Cross-sex full sibling	2 495 461	74 116	2.82%	7.84%	2.89 (2.77 to 3.01)
Cross-sex maternal half-sibling	454 349	27 081	5.76%	9.45%	1.72 (1.61 to 1.83)
Cross-sex paternal half-sibling	581 301	30 420	5.13%	7.39%	1.48 (1.38 to 1.58)

* Models adjusted for birth year of the proband and the relative.

We observed stronger suicide attempt aggregation among same-sex relative pairs than cross-sex pairs. The higher ORs were most pronounced in first-degree relatives, with non-overlapping CIs, though associations in second-degree relatives showed a similar trend (figure 1, panel b; online supplemental Table S7a).

**Figure 2:**
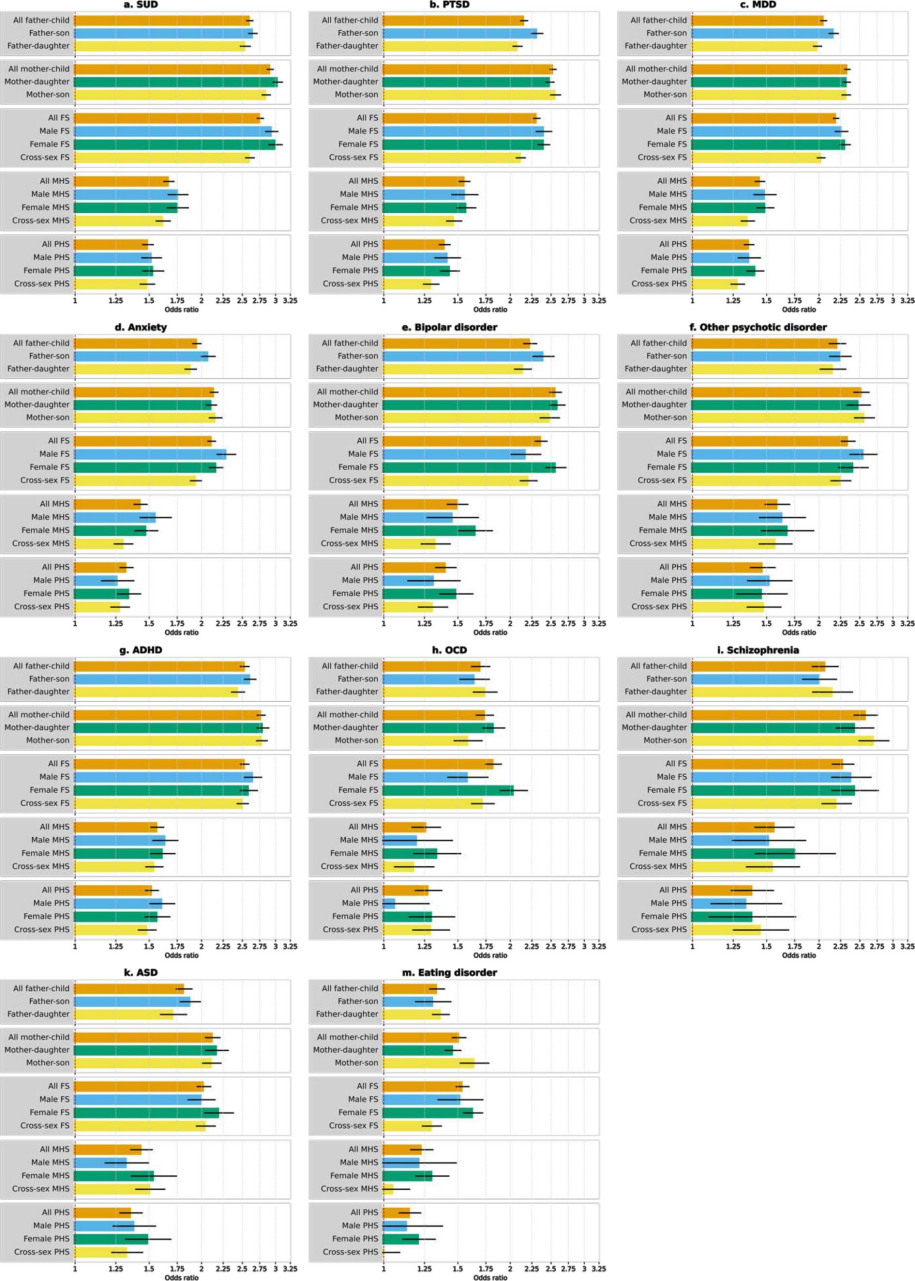
Familial coaggregation of suicide attempt with other psychiatric disorders. Panels (a–m) correspond to coaggregation with 11 psychiatric disorders, as labeled in each subpanel title. Bars show point estimates, error bars show 95% CI for the estimates. Estimated using generalised estimating equations. ADHD, attention deficit/hyperactivity disorder; ASD, autism spectrum disorder; FS, full-siblings; MDD, major depressive disorder; MHS, maternal half-siblings; OCD, obsessive-compulsive disorder; PHS, paternal half-siblings; PTSD, post-traumatic stress disorder; SUD, substance use disorder.

In terms of familial coaggregations, relatives of those who attempted suicide had significantly increased risk for other psychiatric disorders (ORs ranged 1.2–2.9) with higher ORs among the first-degree compared with second-degree relatives (figure 2, online supplemental Table S9a).

The ORs were statistically significantly higher for mother–offspring pairs compared with father–offspring pairs for SUD (ORs 2.9–3.0 vs 2.5–2.6, respectively), PTSD (ORs 2.5–2.6 vs 2.1–2.3, respectively) and MDD (ORs 2.3 vs 2.0–2.2, respectively) (figure 2, panels a-c; online supplemental Table S10a).

Estimates of familial aggregation and coaggregation for self-harm were similar to those for suicidal attempt (online supplemental Tables S7b, S8b, S9b and S10b). As the estimates from the sensitivity analyses of the familial (co)aggregation where suicide attempt was treated as a time-to-event variable aligned with the main results (online supplemental Tables S7 and S9), the heritability and genetic correlation analyses treated suicide attempt as a binary variable.

Furthermore, estimates of familial aggregation when including immigrant families were similar as those based on only Swedish-born populations (online supplemental Table S11).

Across all kinships and both sexes, familial risks varied only modestly by relative’s age at first attempt onset. In most full-sibling and half-sibling pairs with different sex combinations, we observed slightly stronger associations if the relative’s suicidal behaviours occurred earlier in life, particularly for self-harm. However, there is no clear pattern for the parent-offspring pairs (online supplemental Table S12).

### Heritability and genetic correlation with other psychiatric disorders

We included 2 143 644 unique full-sibling pairs and 343 075 unique maternal half-sibling pairs when estimating pedigree heritability and genetic correlations. The intraclass correlations of full siblings were 1.7 to 1.9 times higher than those of maternal half-siblings, consistent with the assumptions of the biometrical models that familial resemblance arises from genetic contributions (online supplemental Table S13).

**Figure 3:**
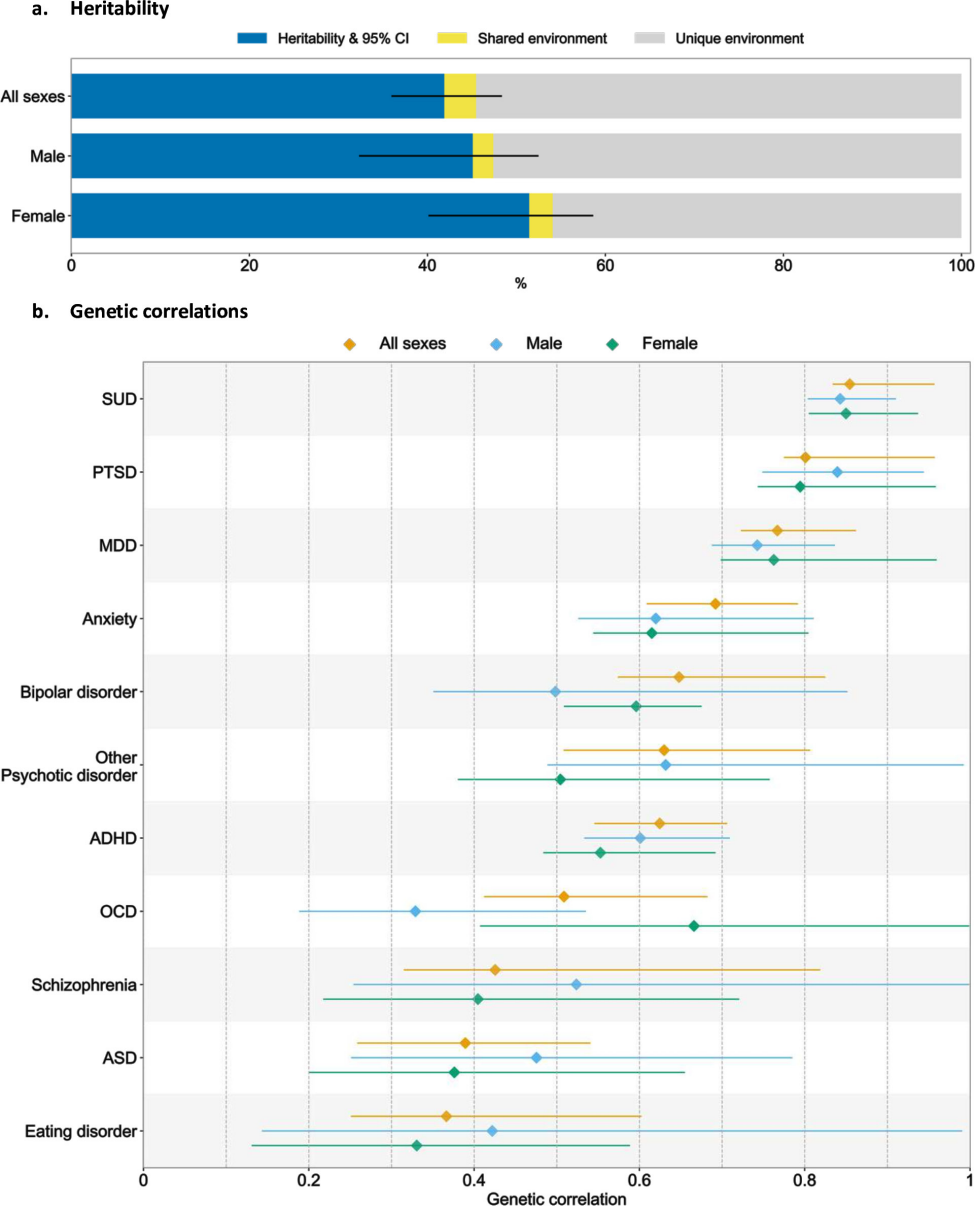
Heritability of suicide attempt (panel a) and genetic correlation of suicide attempt with psychiatric disorders (panel b). Markers show point estimates, error bars show 95% CI for the estimates (full results in online supplemental Table S10 and S12). Estimated using ACE models. ADHD, attention deficit/hyperactivity disorder; ASD, autism spectrum disorder; MDD, major depressive disorder; MHS, maternal half-siblings; OCD, obsessive-compulsive disorder; PTSD, post-traumatic stress disorder; SUD, substance use disorder.

The pedigree-heritability of suicide attempt was 41.9% (95% CI 36.0% to 48.4%). We observed a small but statistically significant influence of shared environmental factors (3.6%, 95% CI 0.7% to 6.3%). Estimated heritability was similar in females (*h*^2^ 51.4%, 95% CI 40.1 to 58.6%) and males (*h*^2^ 45.1%, 95% CI 32.3% to 52.5%); between-sex difference was not statistically significant (p=0.40). The shared environment component was also similar between sexes (figure 3, panel a; online supplemental Table S14a). The genetic correlation between female and male suicide attempt was 0.85 (95% CI 0.80 to 0.99, p value for testing *r_g_*=1: <0.001) (online supplemental Table S15a).

There were moderate to high pedigree genetic correlations between suicide attempt and 11 psychiatric disorders, ranging from 0.37 to 0.85, with the highest correlation noted for SUD (*r_g_*=0.85, 95% CI 0.83 to 0.96) followed by PTSD (*r_g_*=0.80, 95% CI 0.77 to 0.96) and MDD (*r_g_*=0.77, 95% CI 0.72 to 0.86). We did not observe a statistically difference in genetic correlations for males and females (figure 3, panel b; online supplemental Table S15a and S16a).

Estimates of heritability for self-harm were comparable to estimates for suicide attempt (pedigree-heritability 42.3%, 95% CI 37.0% to 47.9%; online supplemental Table S14a). Compared with suicide attempt, the *r_g_* of self-harm with other psychiatric disorders was attenuated by 9%–43% in all analyses (online supplemental Tables S15b and S16b).

## Discussion

Analysing data from a Swedish birth cohort of 3.6 million individuals, we identified a higher incidence of suicide attempt among females than males. Familial risk patterns differed by sex, with higher aggregation of suicide attempt observed in female relatives than male relatives. Notably, the aggregation was higher among same-sex relatives compared with cross-sex relative pairs. However, estimates of heritability and genetic correlations with other psychiatric disorders did not differ significantly by sex. These findings reveal complex genetic and environmental influences on suicide attempt.

We observed clear familial aggregation for suicide attempt, with notable sex differences among first-degree relatives (parents–offspring and full-siblings). Specifically, the familial risks were higher in female dyads compared with male dyads. Similar patterns have been reported in other psychiatric disorders such as ADHD, MDD and schizophrenia, but the evidence has been mixed and inconclusive.^22^,^24^ In this study, we disentangled these complex patterns by examining relationship type, sex pairing (same vs cross-sex) and degree of relatedness.

The increased familial aggregation of suicide attempt among females was limited to first-degree relatives. For example, higher ORs were observed in mother–offspring compared with father–offspring pairs, which may reflect differences in shared environment, including in-utero, early life exposures as well as sex-specific biological factors. Additionally, because suicide attempts are often more fatal in males,^4^ some males may die before becoming a father and not being captured in the dataset, potentially leading to an underestimation of familial risk in father–offspring pairs. Among second-degree relatives, the absence of sex differences in familial risk may be due to smaller shared genetics and less shared environment due to less cohabitation. For example, in Sweden, half-siblings are less likely to live together than full siblings,^20^ potentially contributing to the stronger familial aggregation observed in first-degree relatives.

Among first-degree relatives, we also observed stronger familial aggregation in same-sex dyads compared with opposite-sex dyads, particularly among females (eg, mother–daughter, sister–sister). While previous research has reported a higher risk of suicide attempt among male offspring of parents with a history of attempts,^13^ little attention has been given to the role of same-sex versus cross-sex familial risk. Our findings suggest that sex-matching may amplify the familial risk, which could be partially explained by sex-specific healthcare-seeking behaviours (females are more likely to be admitted for self-harm, raising detection in clinical registers). However, the robustness of findings using stricter phenotype definition suggests that sex differences are not solely artefacts of healthcare-seeking.

Despite notable sex differences in familial risks among first-degree relatives, we found no major difference in the genetic components of suicide attempt. Our heritability estimate was 42% consistent with prior studies using twin and sibling data (online supplemental Table S1), but more robust as we used high-quality register data, capturing severe attempts. Heritability estimate was slightly higher in females than males but not statistically significant (p value=0.40), and the genetic correlation between female and male suicide attempt was high (0.85), suggesting similar underlying genetic influence. Moreover, we showed moderate to high genetic overlap between suicide attempt and other psychiatric disorders, with similar correlation estimates in both females and males. These findings align with a previous Swedish study where suicide attempts and death were analysed separately, and with limited genomic evidence—likely constrained by low case numbers—showing no sex differences in the association between suicide death polygenic risk scores (PRS) and psychiatric traits.^25^

These observations—sex differences in familial risk and incidence but not in genetic components—mirror patterns seen in psychiatric disorders. For example, females with ADHD have higher family history of the disorder than males but show no differences in PRS or copy number variants.^26^ Similarly, while MDD shows sex differences in incidence,^27^ genetic influences largely overlap. Taken together, our findings do not support the hypothesis that genetic factors explain the higher incidence of suicide attempts in females. Non-genetic factors—such as hormonal, neurobiological and environmental interactions—may underlie sex-specific vulnerabilities to suicide attempt.^28^ Future research may focus on sex differences in shared environmental factors, such as adverse childhood experience, and their potential interaction with genetic risk, as a prior sex-stratified genome-wide gene-by-environment study has identified sex-specific risk loci for suicidality.^29^

A key strength of our study is the use of high-quality, population-based longitudinal register data, which allows a comprehensive analysis of sex differences in suicide attempts across familial and genetic contexts. Our study is among the few to provide such an extensive overview in the general population, capturing both first-degree and second-degree relatives and enabling examination of same-sex versus cross-sex dyads. Several limitations merit comment. First, reliance on register data may underestimate suicide attempt incidence by missing cases not attended by specialised care (*eg,* primary care) or resulting in death. Nonetheless, our focus on suicide attempt likely captures the most severe cases identified through specialist care and death records. However, suicidal behaviour severity may correlate with latent genetic liability, the stricter phenotype could inflate heritability estimates in relative to the broader phenotype. Second, the aggregation design is widely used to detect the presence of familial risk factors for suicidal behaviour and comorbid conditions after accounting the birth year and sex difference. However, secular changes in suicide attempt and other psychiatric disorders’ incidence, psychiatric treatment and diagnostic practice over the decades may affect observed associations but could not be adjusted in the study. Third, the generalisability of findings is limited by the inclusion of a relatively young population born in Sweden (and migrating to Sweden in childhood) to ensure complete follow-up and minimise bias in genetic estimates. Environmental distributions, gene–environment interplay and psychiatric care use differ in Swedish-born and foreign-born individuals, our heritability estimates may not transport directly to immigrant population. In particular, if the risk of suicidal behaviour is more environment-loaded among immigrants, excluding them could overestimate the genetic contributions (heritability) at population level. Prior research supports the comparability of Sweden’s healthcare, and psychiatric morbidities and mortality with other high-income countries, supporting the generalisability of our results to such settings.^30^ Finally, our models relied on the assumption of no gene–environment interplay, which, if violated, could impact the estimates. Investigating G × E was not within the scope of this study due to complex methodologies and challenges in measuring specific environment influence. While evidence for G × E remain sparse in psychiatry genetics, this could be an area for future study.

## Conclusions and clinical implications

We found sex differences in familial aggregation of suicide attempts and coaggregation with psychiatric disorders, whereas heritability and genetic correlations were similar across sexes. This suggests that genetic liability contributes to suicide attempt risk but does not fully explain the higher incidence in females, underscoring the role of non-genetic factors and potential gene–environment interplay.

Considering the sex of affected relatives, which is often overlooked in current practice, may help identify individuals at elevated risk of suicide attempt, particularly given stronger familial aggregation in same-sex dyads in women. Although heritability and familial aggregation reflect population-level patterns and should not be interpreted as deterministic or predictive at the individual level, incorporating sex-specific family history into clinical assessment and public health surveillance could strengthen the targeting and evaluation of prevention efforts. Therefore, our findings provide a practical pathway for policymakers to target and evaluate suicide prevention strategies aligned with national and global goals to promote mental health.

## Supplementary material

10.1136/bmjment-2025-302082 Supplementary file 1

## Data Availability

Data may be obtained from a third party and are not publicly available.
