# Supplementary material for: Sex differences in familial risk and genetic components of suicide attempts: a register-based cohort study in Sweden
Source: BMJ Ment Health. 2026 Mar 10;29(1):e302082. doi: 10.1136/bmjment-2025-302082 (PMC12983717; doi:10.1136/bmjment-2025-302082)
Supplement: Supplementary file 1 [file bmjment-29-1-s001.docx]

Supplement for:

**Sex differences in familial risk and genetic components of suicide attempt**

Thuy-Dung Nguyen; Tong Gong; Kejia Hu; Ralf Kuja-Halkola; Karen Borges; Agnieszka Butwicka; Isabell Brikell; James J. Crowley; Zheng Chang; Brian M. D’Onofrio; Henrik Larsson; Paul Lichtenstein; Christian Rück; Cynthia M. Bulik; Fang Fang; Patrick F. Sullivan; Yi Lu

Corresponding authors: Yi Lu (lu.yi@ki.se); Thuy-Dung Nguyen (thuy.dung.nguyen@ki.se)

**Table of Contents**

[SUPPLEMENTARY TABLES 2](#_Toc214611004)

[Table S1: Literature review 2](#_Toc214611005)

[Table S2: Register data 4](#_Toc214611006)

[Table S3: ICD codes of suicide attempts and psychiatric disorders 5](#_Toc214611007)

[Table S4. Overview of methods 7](#_Toc214611008)

[Table S5: Population characteristics 7](#_Toc214611009)

[Table S6: Proportion of individuals with comorbid psychiatric disorders 8](#_Toc214611010)

[Table S7: Familial aggregation 9](#_Toc214611011)

[Table S8: Sex-specific familial aggregation 10](#_Toc214611012)

[Table S9: Familial coaggregation with other psychiatric disorders 12](#_Toc214611013)

[Table S10: Sex-specific familial coaggregation with other psychiatric disorders 16](#_Toc214611014)

[Table S11: Sensitivity analysis on familial risks of suicide attempt and self-harm among extended populations. 24](#_Toc214611015)

[Table S12: Familial aggregation by relative’s age at first attempt and by sex 25](#_Toc214611016)

[Table S13: Intra-class correlations 27](#_Toc214611017)

[Table S14: Heritability 27](#_Toc214611018)

[Table S15: Genetic correlations 28](#_Toc214611019)

[Table S16: Sex-specific genetic correlations 30](#_Toc214611020)

[SUPPLEMENTARY FIGURES 31](#_Toc214611021)

[Figure S1: Sample size 31](#_Toc214611022)

[References 32](#_Toc214611023)

# **SUPPLEMENTARY TABLES**

## **Table S1: Literature review**

| **Author, year** | **Title** | **Population (Sample size)** | **Phenotype** | **Results summary** |
| --- | --- | --- | --- | --- |
| Kendler, 2023^1^ | Genetic liability to suicide attempt, suicide death, and psychiatric and substance use disorders on the risk for suicide attempt and suicide death: a Swedish national study | Individuals born in Sweden during 1932–1995, to Swedish-born parents  (N=7,661,519; 3.27% suicide attempts; 0.57% suicide death) | Suicide attempt (ICD-10: X60-X84, Y10-Y34 and equivalent codes in ICD-8/9) registered in inpatient, outpatient, and primary care | Genetic susceptibilities to suicide attempt and suicide death are related but not identical;  Family genetic risk score (FGRS) for suicide attempt and alcohol use disorder were higher in predicting suicide attempts, while the FGRS for suicide death, bipolar disorder, and schizophrenia were higher in predicting suicide deaths. |
| Edwards, 2021^2^ | On the genetic and environmental relationship between suicide attempt and death by suicide | Twins, full siblings, and half siblings born in Sweden during 1960-1990 (N=1,314,990; 21,664 females and 21,854 males attempted suicide; 1,048 females and 3,109 males died by suicide) | Suicide attempt (ICD-10: X60-X84, Y10-Y34 and equivalent codes in ICD-8/9) registered in inpatient and outpatient | Heritability of suicide attempt: Females 0.52 (95% CI=0.44-0.56)  Males 0.41 (95% CI=0.38-0.49) Heritability of suicide death: Females 0.45 (95% CI=0.39-0.59)  Males 0.44 (95% CI=0.43-0.44)  Genetic correlation between suicide attempt and suicide death: Female 0.67 (95% CI=0.55-0.67)  Male 0.74 (95% CI=0.63-0.87) |
| Kendler, 2020^3^ | The sources of parent-child transmission of risk for suicide attempt and deaths by suicide in Swedish National Samples | Individuals born in Sweden during 1960-1990  (N offspring: 2,175,259 in intact families; 152,436 not lived with father; 73,785 lived with stepfather; 15,624 in adoptive families.  Suicide attempt among offspring: 2.8% in intact families; 6.3% in those not lived with father, 5.5% in those lived with stepfather, 6.7% among those lived in adoptive families) | Suicide attempt (ICD-8/9: E95 and E98; ICD-10: X60–X89, Y10–Y34, Y87, Y90, Y91) registered in inpatient and outpatient | Suicide attempt transmitted through generation Genes only (correlation 0.13, 95% CI 0.11-0.15) Genes plus rearing (correlation 0.23, 95% CI 0.23-0.24) Rearing only (correlation 0.14 (95% CI 0.11-0.16); Suicide attempt was more strongly transmitted to male offspring compared with female offspring; Parental psychiatric disorders accounted for 40% of the genetic transmission but had no impact on rearing effects |
| Cho, 2006^4^ | Genetic contribution to suicidal behaviors and associated risk factors among adolescents in the U.S. | Adolescents in grades 7–12 in the United States from the National Longitudinal Study of Adolescent Health (Add Health)  (N=1448 individuals, 724 twin pairs) | 1 self-reported item in questionnaire | Heritability 24% (calculated from concordance rate using Holzinger’s formula: 3 of 13 MZ pairs and 2 of 21 DZ pairs were concordant; 37.5% versus 17.4%; H_C_ = 24%) |
| Baldessarini, 2004^5^ | Genetics of suicide: an overview | Systematic review included 22 studies (N~25,000 individuals with suicidal behavior and family members) | Compare risks of suicides or of serious attempts among close relatives of index individuals with suicidal behavior, with such risks among relatives of non-suicidal or healthy controls. | Familial risk of suicidal behavior: Combined risk ratio 2.86 (95% CI 2.32–3.53) |
| Fu, 2002^6^ | A twin study of genetic and environmental influences on suicidality in men | Twin pairs from the Vietnam Era Twin Registry who were assessed in 1987 and 1992  (N=3372 twin pairs) | Suicide ideation and suicide attempt from self-reported | Twins whose monozygotic (MZ) co-twin had a record of suicidal ideation showed a higher tendency to report suicidal ideation (OR 2.96) or suicide attempt (OR 5.34).  Twins whose MZ co-twin had a history of suicide attempts had an increased likelihood of reporting suicidal ideation (OR 4.30) or suicide attempt (OR 12.06), compared to twins whose co-twin hadn't reported any suicidal behaviors.  Heritability of suicide attempts: 17.4% (95% CI 13.8–43.7)  Heritability of suicide ideation: 36.0% (95% CI 15.5–41.3) |
| Glowinski, 2001^7^ | Suicide attempts in an adolescent female twin sample | Missouri female adolescent twins, mean age 15.5 years  (N=3,416 individuals; 4.2% with suicide attempts) | Self-reported suicide attempt | Odds ratio for twin/co-twin suicide attempt: 11.6 (95% CI 4.7–28.6) for MZ twins; 4.2 (95% CI 1.2–15.3) for DZ twins  Heritability: 48% (95% CI 0–73.2) |
| Statham, 1998^8^ | Suicidal behaviour: an epidemiological and genetic study | Twins from an Australian twin panel  (N=5995 individuals; 3.0% female and 2% male with suicide attempts) | Self-reported suicide attempt | Heritability: 43% for any suicidal thoughts; 44% for persistent thoughts, plans or minor attempt; and 55% for serious suicide attempt (No CI provided) |

## **Table S2: Register data**

| **Register Name** | **Coverage year** | **Recorded information** |
| --- | --- | --- |
| Total Population Register (TPR)^9^ | Started in 1968;  Covers ~100% population | Dates of birth, sex, marital status |
| Swedish Medical Birth Register^10^ | Started in 1973;  Covers ~98% of births in Sweden | Maternal data (health and lifestyle), birth and neonatal care data;  Used to identify individuals who were born in Sweden |
| National Patient Register (NPR)^11^ | The NPR includes information on hospital-based inpatient healthcare utilization since 1964 and has covered inpatient care of psychiatric disorders nationwide since 1973. From 2001 onward, the NPR also includes information on specialist outpatient care. Covers ~100% for both psychiatric and somatic diseases in 1978 in inpatient care, ~80% for outpatient care (missingness mainly due to not including data from private caregivers) | Inpatient discharges and outpatient visits (Not including primary care records);  Diagnoses pertaining to visit (ICD codes), date of discharge and location of discharge, length of admission, procedures completed. Each entry in the NPR lists a primary diagnosis and up to 30 secondary diagnoses |
| Multi-Generation Register^12^ | Started in 1947;  Covers 97% on mothers, 95% on fathers of index persons | ID of parents of index persons who were born from 1932 onwards and were alive on 1/1/1961 |
| Cause of Death Register^13^ | Started in 1952;  Covers ~100% death in Sweden | The register records the date of death and the underlying and contributing causes of each death, coded according to the ICD system. In Sweden, an apparent or suspected unnatural death typically necessitates a forensic autopsy, which includes toxicological analysis |

##

## **Table S3: ICD codes of suicide attempts and psychiatric disorders**

| **Phenotype** | **ICD 10 (1997-2019)** | **ICD 9 (1987-1996)^a^** | **ICD 8 (1968-1986)** |
| --- | --- | --- | --- |
| **Any self-harm including suicide attempt** | 1. Intentional self-harm/poisoning (ICD-10: X60-X84); 2. Suicide and self-inflicted poisoning/injury (ICD-8/9: E950-E959); 3. Self-harm with undetermined intent (ICD-10: Y10-Y34, ICD-8/9: E980-E989); 4. Sequelae (ICD-10: Y87.0, Y87.2) 5. Death with any X, Y, E codes from Cause of death register | | |
| **Suicide attempt** | 1. X60-X84 2. Any of the other codes (Y10-Y34, Y87.0, Y87.2, E950-E959, E980-E989)   **AND** meeting one of the two additional conditions:   1. Used a notably lethal method of self-harm (firearm, jumping from heights, motor vehicle crash, suffocation, or poisoning by cooking gas) 2. Led to inpatient care 3. Death with any X, Y, E codes from Cause of death register | | |
| **Death by Suicide identified in Cause of death register** | X60-X84, Y10-Y34, Y87.0, Y87.2 | E950-E959 | E950-E959 |
| **Substance use disorders (excluding tobacco) (SUD)** | F10, F11, F12, F13, F14, F15, F16, F18, F19 | 291, 292, 303, 304, 305 (due to truncation of the code, we couldn’t remove 305.1 (305B) which is tobacco abuse | 291, 303, 304 |
| **Schizophrenia, Schizoaffective disorder (SCZ)** | F20.0, F20.1, F20.2, F20.3, F20.4, F20.5, F20.6, F20.8, F20.9, F25.0, F25.1, F25.2, F25.8, F25.9 | 295.0 (295A), 295.1 (295B), 295.2 (295C), 295.3 (295D), 295.4 (295E), 295.6 (295G), 295.8 (295X), 295.9 (295W), 295.7 (295H) | 295.0, 295.1, 295.2, 295.3, 295.4, 295.6, 295.7, 295.8, 295.9 |
| **Other psychotic disorder** | F21, F22, F23, F24, F28, F29 | 297.0 (297A), 297.1 (297B), 297.2 (297C), 297.3 (297D), 297.8 (297X), 297.9 (297W), 298.0 (298A), 298.1 (298B), 298.3 (298D), 298.4 (298E), 298.8 (298W), 298.9 (298X) | 297.0, 297.1, 297.9, 298.0, 298.3, 298.9 |
| **Bipolar, manic disorder** | F30.1, F30.2, F30.8, F30.9, F31 | 296.1 (296D), 296.4 (296E), 296.5 (296F), 296.6 (296G), 296.7 (296H), 296.89 (296W), 296.99 (296X) | 296.1, 296.3, 296.8 |
| **Major depressive disorder (MDD)** | F32, F33 | 296.3 (296B), 311 | 300.4 |
| **Anxiety** | F40.0, F40.1, F40.2, F41.0, F41.1 | 300.0 (300A), 300.2 (300C) | 300.0, 300.2 |
| **Obsessive-compulsive disorder (OCD)** | F42.0, F42.1, F42.2, F42.8, F42.9 | 300.3 (300D) | 300.3 |
| **Post-traumatic stress disorder (PTSD)** | F43.0, F43.1, F43.2, F43.8, F43.9 | 308.0 (308A), 308.1 (308B), 308.2 (308C), 308.3 (308D), 308.4 (308E), 308.9 (308X),  309.0 (309A), 309.1 (309B), 309.2 (309C), 309.3 (309D), 309.4 (309E), 309.8 (309W), 309.9 (309X) | 307 |
| **Eating disorder** | F50.0, F50.1, F50.2, F50.3, F50.4, F50.5, F50.8, F50.9 | 307.1 (307B), 307.5 (307F) | N/A |
| **Autism spectrum disorder (ASD)** | F84.0, F84.1, F84.5 | 299.0 (299A) | N/A |
| **Attention deficit hyperactivity disorder (ADHD)** | F90.0, F90.1, F90.8 | 314.01 (314, 314A), 314.1 (314B), 314.2 (314C, 314J), 314.8 (314W), 314.9 (314X) | N/A |

*If the last characters of the code were not specified, all codes starting with the specified characters were included*

*^a^Swedish version of the code, if different from the international codes, is specified in brackets*

## **Table S4. Overview of methods**

| **Analyses** | **Estimates/measurements** | **Statistical methods** |
| --- | --- | --- |
| Familial risks | Familial aggregation of suicide attempt, and coaggregation of suicide attempt with 11 psychiatric disorders; odds ratio (OR) | - Generalized Estimating Equations with logit link function - Parent-offspring, full-siblings, half-siblings |
| Pedigree heritability (*h^2^*) | *h^2^*of suicide attempt | - Structural equation modelling - Confidence interval from Bootstrap resampling - Comparing full-siblings and maternal-half siblings |
| Genetic correlation (*r_g_)* | *r_g_* between male and female suicide attempt  *r_g_* between suicide attempt and 11 psychiatric disorders |  |

## **Table S5: Population characteristics**

| **Characteristics** | **All population**  **N=3,058,374** | **Male**  **N=1,571,720** | **Female**  **1,486,654** |
| --- | --- | --- | --- |
| **Age at end of follow-up** |  |  |  |
| Mean (SD) | 39.61 (10.48) | 39.61 (10.48) | 39.61 (10.48) |
| Range | 21.04-56.96 | 21.04-56.96 | 21.04-56.96 |
| Median | 39.79 | 39.79 | 39.79 |
| 1^st^ quartile-3^rd^ quartile | 30.29-48.87 | 30.29-48.87 | 30.29-48.87 |
| **Suicide attempt** (N, %) | 89278 (2.92%) | 40154 (2.56%) | 49124 (3.30%) |
| **Self-harm** (N, %) | 126411 (4.13%) | 63624 (4.05%) | 62787 (4.22%) |

## **Table S6: Proportion of individuals with comorbid psychiatric disorders**

|  | **Both sexes** | | **Female** | | **Male** | |
| --- | --- | --- | --- | --- | --- | --- |
| **Psychiatric disorder** | **Without suicide attempt**  **(N=2,969,096)** | **With suicide attempt**  **(N=89,278)** | **Without suicide attempt**  **(N=1,437,530)** | **With suicide attempt**  **(N=49,124)** | **Without suicide attempt**  **(N=1,531,566)** | **With suicide attempt**  **(N=40,154)** |
| SUD | 124544  (4.19%) | 41530  (46.52%) | 46563  (3.24%) | 21250  (43.26%) | 77981  (5.09%) | 20280  (50.51%) |
| MDD | 187021 (6.30%) | 41363 (46.33%) | 111910  (7.78%) | 26241  (53.42%) | 75111  (4.90%) | 15122  (37.66%) |
| PTSD | 121234 (4.08%) | 29497 (33.04%) | 78412  (5.45%) | 19383  (39.46%) | 42822  (2.8%) | 10114  (25.19%) |
| Anxiety | 88873  (2.99%) | 16907 (18.94%) | 54551  (3.79%) | 10914  (22.22%) | 34322  (2.24) | 5993  (14.93%) |
| ADHD | 84389  (2.84%) | 16718 (18.73%) | 35158  (2.45%) | 8866  (18.05%) | 49231  (3.21%) | 7852  (19.55%) |
| Bipolar disorder | 30304  (1.02%) | 10513 (11.78%) | 18732  (1.30%) | 7211  (14.68%) | 11572  (0.76%) | 3302  (8.22%) |
| Other psychotic disorders | 19126 (0.64%) | 7544 (8.45%) | 7520  (0.52%) | 3489  (7.10%) | 11606  (0.76%) | 4055  (10.10%) |
| Eating disorder | 43811 (1.48%) | 7004 (7.85%) | 35452  (2.47%) | 6172  (12.56%) | 8359  (0.55%) | 832  (2.07%) |
| ASD | 33290 (1.12%) | 5427 (6.08%) | 11693  (0.81%) | 2989  (6.08%) | 21597  (1.41%) | 2438  (6.07%) |
| OCD | 24188 (0.81%) | 4164 (4.66%) | 13973  (0.97%) | 2830  (5.76%) | 10215  (0.67%) | 1334  (3.32%) |
| Schizophrenia | 8129  (0.27%) | 3487 (3.91%) | 2866  (0.20%) | 1641  (3.34%) | 5263  (0.34%) | 1846  (4.60%) |
| Any of above 11 disorders | 451092 (15.19%) | 67527 (75.64%) | 240352  (16.72%) | 38826  (79.04%) | 210740  (13.76%) | 28701  (71.48%) |

*SUD: Substance use disorder | MDD: Major depressive disorder | PTSD: Post-traumatic stress disorder | ADHD: Attention deficit hyperactivity disorder | ASD: Autism spectrum disorder | OCD: Obsessive-compulsive disorder*

## **Table S7: Familial aggregation**

| - - - - 1. **Suicide attempt** | | | | | | | | | | | | | | |
| --- | --- | --- | --- | --- | --- | --- | --- | --- | --- | --- | --- | --- | --- | --- |
|  | **Generalised Estimating Equations** | | | | | | **Cox regression** | | | | | | | |
| **Relative types** | **Coefficient** | **SE** | **p-value** | **OR** | **95% CI of OR** | | **Coefficient** | **SE** | **p-value** | | **OR** | | **95% CI of HR** | |
| Mother-offspring | 1.2130 | 0.0133 | <2.2*10^-308^ | 3.3635 | 3.2772 | 3.4522 | 1.2037 | 0.0131 | <2.2*10^-308^ | 3.3323 | | 3.2477 | | 3.4191 |
| Father-offspring | 1.0384 | 0.0140 | <2.2*10^-308^ | 2.8246 | 2.7482 | 2.9032 | 1.0630 | 0.0141 | <2.2*10^-308^ | 2.8950 | | 2.8163 | | 2.9758 |
| Full sibling | 1.1749 | 0.0153 | <2.2*10^-308^ | 3.2379 | 3.1420 | 3.3367 | 1.1179 | 0.0148 | <2.2*10^-308^ | 3.0584 | | 2.9710 | | 3.1485 |
| Maternal half-sibling | 0.5890 | 0.0244 | 3.5*10^-129^ | 1.8022 | 1.7182 | 1.8903 | 0.5467 | 0.0235 | 2.4*10^-119^ | 1.7275 | | 1.6496 | | 1.8091 |
| Paternal half-sibling | 0.4557 | 0.0249 | 1.2*10^-74^ | 1.5773 | 1.5021 | 1.6563 | 0.3975 | 0.0247 | 1.7*10^-58^ | 1.4881 | | 1.4179 | | 1.5618 |

| - - - - 1. **Self-harm** | | | | | | | | | | | | | | |
| --- | --- | --- | --- | --- | --- | --- | --- | --- | --- | --- | --- | --- | --- | --- |
|  | **Generalised Estimating Equations** | | | | | | **Cox regression** | | | | | | | |
| **Relatedness** | **Coefficient** | **SE** | **p-value** | **OR** | **95% CI of OR** | | **Coefficient** | **SE** | **p-value** | | **OR** | | **95% CI of HR** | |
| Mother-offspring | 1.1035 | 0.0111 | <2.2*10^-308^ | 3.0147 | 2.9500 | 3.0808 | 1.0542 | 0.0111 | <2.2*10^-308^ | 2.8697 | | 2.8076 | | 2.9330 |
| Father-offspring | 1.0134 | 0.0112 | <2.2*10^-308^ | 2.7549 | 2.6949 | 2.8162 | 0.9750 | 0.0115 | <2.2*10^-308^ | 2.6513 | | 2.5923 | | 2.7116 |
| Full sibling | 1.0957 | 0.0119 | <2.2*10^-308^ | 2.9914 | 2.9223 | 3.0622 | 1.0253 | 0.0115 | <2.2*10^-308^ | 2.7878 | | 2.7256 | | 2.8515 |
| Maternal half-sibling | 0.5675 | 0.0205 | 2.8*10^-168^ | 1.7639 | 1.6943 | 1.8363 | 0.5195 | 0.0199 | 1.3*10^-150^ | 1.6812 | | 1.6170 | | 1.7480 |
| Paternal half-sibling | 0.4148 | 0.0209 | 6.1*10^-88^ | 1.5141 | 1.4534 | 1.5773 | 0.3638 | 0.0205 | 2.2*10^-70^ | 1.4388 | | 1.3821 | | 1.4978 |

*Note: Estimates for all sexes*

## **Table S8:** **Sex-specific familial aggregation**

| **Suicide attempt** | | | | | | | | | | | | | |
| --- | --- | --- | --- | --- | --- | --- | --- | --- | --- | --- | --- | --- | --- |
|  | **Male** | | | | | | **Female** | | | | | | |
| **Relative types** | **Coefficient** | **SE** | **p-value** | **OR** | **95% CI of OR** | | **Relative types** | **Coefficient** | **SE** | **p-value** | **OR** | **95% CI of OR** | |
| Father-son | 1.1055 | 0.0195 | <2.2*10^-308^ | 3.0208 | 2.9073 | 3.1387 | Mother-daughter | 1.2483 | 0.0177 | <2.2*10^-308^ | 3.4844 | 3.3659 | 3.6070 |
| Male full sibling | 1.2113 | 0.0306 | <2.2*10^-308^ | 3.3578 | 3.1622 | 3.5654 | Female full sibling | 1.2988 | 0.0276 | <2.2*10^-308^ | 3.6649 | 3.4722 | 3.8683 |
| Male maternal half-sibling | 0.5897 | 0.0511 | 8.0*10^-31^ | 1.8034 | 1.6316 | 1.9934 | Female maternal half-sibling | 0.6294 | 0.0427 | 4.0*10^-49^ | 1.8765 | 1.7258 | 2.0404 |
| Male paternal half-sibling | 0.4874 | 0.0495 | 6.5*10^-23^ | 1.6281 | 1.4777 | 1.7938 | Female paternal half-sibling | 0.5031 | 0.0428 | 7.1*10^-32^ | 1.6538 | 1.5207 | 1.7986 |
|  | **Cross-sex** | | | | | |  |  |  |  |  |  |  |
| **Relatedness** | **Coefficient** | **SE** | **p-value** | **OR** | **95% CI of OR** | |  |  |  |  |  |  |  |
| Mother-son | 1.1660 | 0.0193 | <2.2*10^-308^ | 3.2093 | 3.0900 | 3.3332 |  |  |  |  |  |  |  |
| Father-daughter | 0.9841 | 0.0192 | <2.2*10^-308^ | 2.6754 | 2.5767 | 2.7780 |  |  |  |  |  |  |  |
| Cross-sex full sibling | 1.0598 | 0.0209 | <2.2*10^-308^ | 2.8857 | 2.7699 | 3.0063 |  |  |  |  |  |  |  |
| Cross-sex maternal half-sibling | 0.5413 | 0.0334 | 3.2*10^-59^ | 1.7182 | 1.6094 | 1.8343 |  |  |  |  |  |  |  |
| Cross-sex paternal half-sibling | 0.3904 | 0.0351 | 1.1*10^-28^ | 1.4776 | 1.3793 | 1.5829 |  |  |  |  |  |  |  |

| - 1. **Self-harm** | | | | | | | | | | | | | |
| --- | --- | --- | --- | --- | --- | --- | --- | --- | --- | --- | --- | --- | --- |
|  | **Male** | | | | | | **Female** | | | | | | |
| **Relative types** | **Coefficient** | **SE** | **p-value** | **OR** | **95% CI of OR** | | **Relatedness** | **Coefficient** | **SE** | **p-value** | **OR** | **95% CI of OR** | |
| Father-son | 1.0878 | 0.0149 | <2.2*10^-308^ | 2.9678 | 2.8823 | 3.0559 | Mother-daughter | 1.1342 | 0.0151 | <2.2*10^-308^ | 3.1086 | 3.0179 | 3.2021 |
| Male full sibling | 1.1928 | 0.0217 | <2.2*10^-308^ | 3.2965 | 3.1594 | 3.4394 | Female full sibling | 1.1742 | 0.0232 | <2.2*10^-308^ | 3.2354 | 3.0919 | 3.3857 |
| Male maternal half-sibling | 0.5856 | 0.0402 | 3.8*10^-48^ | 1.7961 | 1.6601 | 1.9432 | Female maternal half-sibling | 0.6129 | 0.0386 | 9.1*10^-57^ | 1.8457 | 1.7112 | 1.9908 |
| Male paternal half-sibling | 0.4477 | 0.0412 | 1.6*10^-27^ | 1.5647 | 1.4433 | 1.6962 | Female paternal half-sibling | 0.478 | 0.0377 | 6.6*10^-37^ | 1.6129 | 1.4981 | 1.7365 |
|  | **Cross-sex** | | | | | |  |  |  |  |  |  |  |
| **Relative types** | **Coefficient** | **SE** | **p-value** | **OR** | **95% CI of OR** | |  |  |  |  |  |  |  |
| Mother-Son | 1.0730 | 0.0153 | <2.2*10^-308^ | 2.9240 | 2.8379 | 3.0128 |  |  |  |  |  |  |  |
| Father-Daughter | 0.9357 | 0.0158 | <2.2*10^-308^ | 2.5491 | 2.4712 | 2.6294 |  |  |  |  |  |  |  |
| Cross-sex full sibling | 1.0011 | 0.0162 | <2.2*10^-308^ | 2.7213 | 2.6361 | 2.8091 |  |  |  |  |  |  |  |
| Cross-sex maternal half-sibling | 0.5281 | 0.0277 | 4.9*10^-81^ | 1.6956 | 1.6060 | 1.7902 |  |  |  |  |  |  |  |
| Cross-sex paternal half-sibling | 0.3590 | 0.0290 | 2.8*10^-35^ | 1.4320 | 1.3529 | 1.5156 |  |  |  |  |  |  |  |

*Note: Estimated using Generalised Estimating Equations*

## **Table S9:** **Familial coaggregation with other psychiatric disorders**

- - - - 1. **Suicide attempt**

| **Relative types** | **Generalised Estimating Equations** | | | | | | **Cox regression** | | | | | | | | | |
| --- | --- | --- | --- | --- | --- | --- | --- | --- | --- | --- | --- | --- | --- | --- | --- | --- |
|  | **Coefficient** | **SE** | **p-value** | **OR** | **95% CI of OR** | | **Coefficient** | **SE** | | **p-value** | | | **HR** | | **95% CI of HR** | |
| With **substance use disorder** | | | | | | | | | | | | | | | | |
| Mother-offspring | 1.0690 | 0.0100 | <2.2*10^-308^ | 2.9125 | 2.8558 | 2.9703 | 1.1553 | | 0.0138 | | <2.2*10^-308^ | 3.1751 | | 3.0904 | | 3.2622 |
| Father-offspring | 0.9573 | 0.0102 | <2.2*10^-308^ | 2.6046 | 2.5530 | 2.6573 | 1.0014 | | 0.0107 | | <2.2*10^-308^ | 2.7221 | | 2.6654 | | 2.7800 |
| Full sibling | 1.0149 | 0.0103 | <2.2*10^-308^ | 2.7591 | 2.7040 | 2.8154 | 0.9962 | | 0.0134 | | <2.2*10^-308^ | 2.7079 | | 2.6379 | | 2.7799 |
| Maternal half-sibling | 0.5139 | 0.0157 | 8.2*10^-234^ | 1.6719 | 1.6211 | 1.7242 | 0.4802 | | 0.0209 | | 1.6*10^-116^ | 1.6163 | | 1.5514 | | 1.6840 |
| Paternal half-sibling | 0.3996 | 0.0164 | 2.2*10^-131^ | 1.4912 | 1.4441 | 1.5398 | 0.3615 | | 0.0221 | | 3.1*10^-60^ | 1.4355 | | 1.3747 | | 1.4990 |
| With **major depressive disorder** | | | | | | | | | | | | | | | | |
| Mother-offspring | 0.8488 | 0.0090 | <2.2*10^-308^ | 2.3369 | 2.2959 | 2.3786 | 0.8573 | | 0.0122 | | <2.2*10^-308^ | 2.3569 | | 2.3013 | | 2.4137 |
| Father-offspring | 0.7189 | 0.0098 | <2.2*10^-308^ | 2.0522 | 2.0131 | 2.0920 | 0.7478 | | 0.0147 | | <2.2*10^-308^ | 2.1124 | | 2.0523 | | 2.1742 |
| Full sibling | 0.7864 | 0.0091 | <2.2*10^-308^ | 2.1955 | 2.1568 | 2.2349 | 0.7263 | | 0.0136 | | <2.2*10^-308^ | 2.0674 | | 2.0131 | | 2.1232 |
| Maternal half-sibling | 0.3696 | 0.0146 | 5.4*10^-141^ | 1.4472 | 1.4063 | 1.4893 | 0.3034 | | 0.0229 | | 4.5*10^-40^ | 1.3545 | | 1.2950 | | 1.4166 |
| Paternal half-sibling | 0.3100 | 0.0149 | 1.2*10^-95^ | 1.3634 | 1.3241 | 1.4039 | 0.2630 | | 0.0237 | | 1.5*10^-28^ | 1.3009 | | 1.2417 | | 1.3628 |
| With **anxiety** |  |  |  |  |  |  |  | |  | |  |  | |  | |  |
| Mother-offspring | 0.7613 | 0.0125 | <2.2*10^-308^ | 2.1411 | 2.0893 | 2.1942 | 0.7652 | | 0.0192 | | <2.2*10^-308^ | 2.1493 | | 2.0699 | | 2.2318 |
| Father-offspring | 0.6684 | 0.0136 | <2.2*10^-308^ | 1.9511 | 1.8996 | 2.0039 | 0.7343 | | 0.0254 | | 3.1*10^-184^ | 2.0841 | | 1.9830 | | 2.1903 |
| Full sibling | 0.7485 | 0.0123 | <2.2*10^-308^ | 2.1139 | 2.0634 | 2.1655 | 0.6965 | | 0.0199 | | 1.6*10^-269^ | 2.0067 | | 1.9301 | | 2.0864 |
| Maternal half-sibling | 0.3591 | 0.0198 | 3.4*10^-73^ | 1.4320 | 1.3774 | 1.4888 | 0.3129 | | 0.0316 | | 4.4*10^-23^ | 1.3674 | | 1.2852 | | 1.4548 |
| Paternal half-sibling | 0.2817 | 0.0198 | 9.4*10^-46^ | 1.3254 | 1.2749 | 1.378 | 0.2495 | | 0.0326 | | 2.1*10^-14^ | 1.2834 | | 1.2039 | | 1.3682 |
| With **OCD** | | | | | | | | | | | | | | | | |
| Mother-offspring | 0.554 | 0.0254 | 4.2*10^-105^ | 1.7402 | 1.6555 | 1.8291 | 0.6111 | | 0.0554 | | 3.0*10^-28^ | 1.8425 | | 1.6528 | | 2.0540 |
| Father-offspring | 0.5302 | 0.0271 | 5.2*10^-85^ | 1.6993 | 1.6113 | 1.7921 | 0.7379 | | 0.0713 | | 4.0*10^-25^ | 2.0915 | | 1.8189 | | 2.4050 |
| Full sibling | 0.6011 | 0.0237 | 9.8*10^-142^ | 1.8241 | 1.7412 | 1.9108 | 0.4847 | | 0.0385 | | 2.0*10^-36^ | 1.6236 | | 1.5058 | | 1.7508 |
| Maternal half-sibling | 0.2328 | 0.0415 | 2.0*10^-8^ | 1.2621 | 1.1635 | 1.369 | 0.165 | | 0.0683 | | 1.6*10^-2^ | 1.1794 | | 1.0315 | | 1.3484 |
| Paternal half-sibling | 0.2452 | 0.0387 | 2.3*10^-10^ | 1.2778 | 1.1845 | 1.3785 | 0.1928 | | 0.0695 | | 5.5*10^-3^ | 1.2126 | | 1.0583 | | 1.3895 |
| With **PTSD** | | | | | | | | | | | | | | | | |
| Mother-offspring | 0.9272 | 0.0101 | <2.2*10^-308^ | 2.5275 | 2.4779 | 2.5780 | 0.9366 | | 0.0147 | | <2.2*10^-308^ | 2.5512 | | 2.4786 | | 2.6260 |
| Father-offspring | 0.7691 | 0.0113 | <2.2*10^-308^ | 2.1578 | 2.1106 | 2.2060 | 0.8292 | | 0.0189 | | <2.2*10^-308^ | 2.2915 | | 2.2080 | | 2.3781 |
| Full sibling | 0.8379 | 0.0105 | <2.2*10^-308^ | 2.3115 | 2.2645 | 2.3594 | 0.8606 | | 0.0166 | | <2.2*10^-308^ | 2.3645 | | 2.2886 | | 2.4429 |
| Maternal half-sibling | 0.4424 | 0.0166 | 4.6*10^-156^ | 1.5565 | 1.5066 | 1.6080 | 0.3991 | | 0.0261 | | 1.1*10^-52^ | 1.4904 | | 1.4160 | | 1.5688 |
| Paternal half-sibling | 0.3338 | 0.0167 | 7.4*10^-89^ | 1.3963 | 1.3513 | 1.4427 | 0.2853 | | 0.0269 | | 2.6*10^-26^ | 1.3301 | | 1.2619 | | 1.4021 |
| With **eating disorder** | | | | | | | | | | | | | | | | |
| Mother-offspring | 0.4125 | 0.0205 | 4.7*10^-90^ | 1.5107 | 1.4512 | 1.5726 | 0.4947 | | 0.0423 | | 1.3*10^-31^ | 1.6401 | | 1.5096 | | 1.7818 |
| Father-offspring | 0.292 | 0.0225 | 2.3*10^-38^ | 1.3391 | 1.2812 | 1.3996 | 0.146 | | 0.0674 | | 3.0*10^-2^ | 1.1572 | | 1.0139 | | 1.3207 |
| Full sibling | 0.4317 | 0.0194 | 2.5*10^-109^ | 1.5398 | 1.4823 | 1.5996 | 0.3483 | | 0.0279 | | 8.8*10^-36^ | 1.4167 | | 1.3413 | | 1.4963 |
| Maternal half-sibling | 0.2079 | 0.0327 | 2.1*10^-10^ | 1.2311 | 1.1546 | 1.3126 | 0.1187 | | 0.0507 | | 1.9*10^-2^ | 1.1260 | | 1.0194 | | 1.2438 |
| Paternal half-sibling | 0.1434 | 0.0317 | 5.9*10^-6^ | 1.1542 | 1.0847 | 1.228 | 0.0904 | | 0.0506 | | 7.4*10^-2^ | 1.0946 | | 0.9912 | | 1.2088 |
| With **schizophrenia** | | | | | | | | | | | | | | | | |
| Mother-offspring | 0.9497 | 0.0343 | 4.8*10^-169^ | 2.5850 | 2.4170 | 2.7646 | 0.8600 | | 0.0463 | | 4.8*10^-77^ | 2.3633 | | 2.1583 | | 2.5877 |
| Father-offspring | 0.7275 | 0.0377 | 3.7*10^-83^ | 2.0699 | 1.9226 | 2.2284 | 0.9556 | | 0.0536 | | 3.9*10^-71^ | 2.6003 | | 2.3410 | | 2.8882 |
| Full sibling | 0.8249 | 0.0319 | 2.8*10^-147^ | 2.2816 | 2.1432 | 2.4289 | 0.7713 | | 0.0434 | | 1.5*10^-70^ | 2.1627 | | 1.9862 | | 2.3548 |
| Maternal half-sibling | 0.4498 | 0.0568 | 2.4*10^-15^ | 1.5679 | 1.4028 | 1.7525 | 0.4319 | | 0.0748 | | 7.9*10^-9^ | 1.5402 | | 1.3301 | | 1.7836 |
| Paternal half-sibling | 0.3282 | 0.0605 | 6.0*10^-8^ | 1.3884 | 1.2330 | 1.5633 | 0.2306 | | 0.0790 | | 3.5*10^-3^ | 1.2593 | | 1.0786 | | 1.4703 |
| With **bipolar disorder** | | | | | | | | | | | | | | | | |
| Mother-offspring | 0.9410 | 0.0179 | <2.2*10^-308^ | 2.5625 | 2.4742 | 2.6540 | 0.7936 | | 0.0267 | | 2.5*10^-194^ | 2.2113 | | 2.0986 | | 2.3301 |
| Father-offspring | 0.8015 | 0.0199 | <2.2*10^-308^ | 2.2289 | 2.1438 | 2.3174 | 0.6962 | | 0.0335 | | 6.9*10^-96^ | 2.0061 | | 1.8786 | | 2.1423 |
| Full sibling | 0.8620 | 0.0181 | <2.2*10^-308^ | 2.3680 | 2.2854 | 2.4535 | 0.7263 | | 0.0313 | | 2.4*10^-119^ | 2.0674 | | 1.9445 | | 2.1981 |
| Maternal half-sibling | 0.4039 | 0.0303 | 1.8*10^-40^ | 1.4977 | 1.4113 | 1.5894 | 0.2771 | | 0.0536 | | 2.4*10^-7^ | 1.3193 | | 1.1877 | | 1.4654 |
| Paternal half-sibling | 0.3397 | 0.0303 | 4.4*10^-29^ | 1.4045 | 1.3234 | 1.4906 | 0.2778 | | 0.0549 | | 4.2*10^-7^ | 1.3203 | | 1.1856 | | 1.4703 |
| With **other psychotic disorders (excl. SCZ)** | | | | | | | | | | | | | | | | |
| Mother-offspring | 0.9256 | 0.0227 | <2.2*10^-308^ | 2.5234 | 2.4137 | 2.638 | 0.7647 | | 0.0286 | | 2.5*10^-157^ | 2.1483 | | 2.0311 | | 2.2722 |
| Father-offspring | 0.7937 | 0.0244 | 1.1*10^-231^ | 2.2117 | 2.1083 | 2.3201 | 0.8835 | | 0.0328 | | 5.3*10^-160^ | 2.4193 | | 2.2687 | | 2.5798 |
| Full sibling | 0.8518 | 0.0214 | <2.2*10^-308^ | 2.3438 | 2.2473 | 2.4444 | 0.7801 | | 0.0318 | | 2.9*10^-133^ | 2.1817 | | 2.0500 | | 2.3218 |
| Maternal half-sibling | 0.4656 | 0.0357 | 8.3*10^-39^ | 1.5930 | 1.4852 | 1.7085 | 0.4217 | | 0.0527 | | 1.3*10^-15^ | 1.5246 | | 1.3749 | | 1.6905 |
| Paternal half-sibling | 0.3845 | 0.0362 | 2.1*10^-26^ | 1.4689 | 1.3684 | 1.5768 | 0.2874 | | 0.0522 | | 3.7*10^-8^ | 1.3330 | | 1.2033 | | 1.4767 |
| With **autism spectrum disorder** | | | | | | | | | | | | | | | | |
| Mother-offspring | 0.7543 | 0.0217 | 3.9*10^-265^ | 2.1261 | 2.0376 | 2.2184 | 0.8251 | | 0.0612 | | 1.8*10^-41^ | 2.2821 | | 2.0243 | | 2.5728 |
| Father-offspring | 0.5972 | 0.0238 | 7.3*10^-139^ | 1.8169 | 1.7341 | 1.9037 | 0.6377 | | 0.0790 | | 6.7*10^-16^ | 1.8922 | | 1.6208 | | 2.2089 |
| Full sibling | 0.7057 | 0.0206 | 3.6*10^-256^ | 2.0252 | 1.9449 | 2.1088 | 0.5595 | | 0.0320 | | 2.7*10^-68^ | 1.7497 | | 1.6433 | | 1.8631 |
| Maternal half-sibling | 0.3639 | 0.0318 | 2.5*10^-30^ | 1.4390 | 1.3520 | 1.5315 | 0.2420 | | 0.0491 | | 8.4*10^-7^ | 1.2738 | | 1.1569 | | 1.4025 |
| Paternal half-sibling | 0.3065 | 0.0328 | 8.8*10^-21^ | 1.3587 | 1.2741 | 1.4488 | 0.3006 | | 0.0503 | | 2.2*10^-9^ | 1.3507 | | 1.2240 | | 1.4906 |
| With **ADHD** | | | | | | | | | | | | | | | | |
| Mother-offspring | 1.0196 | 0.0127 | <2.2*10^-308^ | 2.7720 | 2.7038 | 2.8420 | 0.9682 | | 0.0308 | | 1.5*10^-216^ | 2.6331 | | 2.4787 | | 2.7971 |
| Father-offspring | 0.9297 | 0.0136 | <2.2*10^-308^ | 2.5339 | 2.4674 | 2.6022 | 1.0219 | | 0.0347 | | 9.6*10^-191^ | 2.7784 | | 2.5958 | | 2.9739 |
| Full sibling | 0.9296 | 0.0132 | <2.2*10^-308^ | 2.5334 | 2.4688 | 2.5997 | 0.7466 | | 0.0212 | | 4.1*10^-271^ | 2.1099 | | 2.0239 | | 2.1995 |
| Maternal half-sibling | 0.4506 | 0.0193 | 5.5*10^-120^ | 1.5693 | 1.5109 | 1.6300 | 0.3451 | | 0.0288 | | 3.9*10^-33^ | 1.4122 | | 1.3347 | | 1.4941 |
| Paternal half-sibling | 0.4216 | 0.0197 | 4.4*10^-102^ | 1.5243 | 1.4667 | 1.5842 | 0.3791 | | 0.0297 | | 3.0*10^-37^ | 1.4609 | | 1.3783 | | 1.5486 |

- - - - 1. **Self-harm**

| **Relative types** | **Generalised Estimating Equations** | | | | | | **Cox regression** | | | | | |  |
| --- | --- | --- | --- | --- | --- | --- | --- | --- | --- | --- | --- | --- | --- |
|  | **Coefficient** | **SE** | **p-value** | **OR** | **95% CI of OR** | | **Coefficient** | **SE** | **p-value** | **HR** | **95% CI of HR** | |  |
| With **substance use disorder** | | | | | | | | | | | | | |
| Mother-offspring | 0.9215 | 0.0094 | <2.2*10^-308^ | 2.5131 | 2.4671 | 2.5600 | 0.9317 | 0.0125 | <2.2*10^-308^ | 2.5388 | 2.4773 | 2.6019 |  |
| Father-offspring | 0.8002 | 0.0095 | <2.2*10^-308^ | 2.2261 | 2.1850 | 2.2679 | 0.7982 | 0.0096 | <2.2*10^-308^ | 2.2215 | 2.1799 | 2.2638 |  |
| Full sibling | 0.8336 | 0.0093 | <2.2*10^-308^ | 2.3015 | 2.2599 | 2.3439 | 0.7840 | 0.0119 | <2.2*10^-308^ | 2.1902 | 2.1396 | 2.2420 |  |
| Maternal half-sibling | 0.4552 | 0.0144 | 2.9*10^-219^ | 1.5764 | 1.5325 | 1.6215 | 0.4036 | 0.0187 | 3.1*10^-103^ | 1.4971 | 1.4432 | 1.5530 |  |
| Paternal half-sibling | 0.3469 | 0.0148 | 3.2*10^-121^ | 1.4147 | 1.3742 | 1.4564 | 0.2847 | 0.0194 | 9.8*10^-49^ | 1.3293 | 1.2797 | 1.3809 |  |
| With **major depressive disorder** | | | | | | | | | | | | |  |
| Mother-offspring | 0.7172 | 0.0084 | <2.2*10^-308^ | 2.0486 | 2.0153 | 2.0825 | 0.6530 | 0.0108 | <2.2*10^-308^ | 1.9213 | 1.8810 | 1.9626 |  |
| Father-offspring | 0.5853 | 0.0090 | <2.2*10^-308^ | 1.7956 | 1.7640 | 1.8277 | 0.5694 | 0.0130 | <2.2*10^-308^ | 1.7672 | 1.7228 | 1.8127 |  |
| Full sibling | 0.6250 | 0.0081 | <2.2*10^-308^ | 1.8682 | 1.8387 | 1.8982 | 0.5202 | 0.0119 | <2.2*10^-308^ | 1.6824 | 1.6438 | 1.7220 |  |
| Maternal half-sibling | 0.3119 | 0.0135 | 1.4*10^-118^ | 1.3660 | 1.3304 | 1.4026 | 0.2395 | 0.0203 | 5.3*10^-32^ | 1.2706 | 1.2209 | 1.3222 |  |
| Paternal half-sibling | 0.2558 | 0.0136 | 2.1*10^-78^ | 1.2915 | 1.2574 | 1.3265 | 0.1868 | 0.0208 | 3.1*10^-19^ | 1.2053 | 1.1571 | 1.2556 |  |
| With **anxiety** | | | | | | | | | | | | |  |
| Mother-offspring | 0.6464 | 0.0117 | <2.2*10^-308^ | 1.9086 | 1.8654 | 1.9528 | 0.5898 | 0.0171 | 5.1*10^-260^ | 1.8035 | 1.7440 | 1.8651 |  |
| Father-offspring | 0.5468 | 0.0126 | <2.2*10^-308^ | 1.7278 | 1.6856 | 1.7710 | 0.5647 | 0.0224 | 1.0*10^-139^ | 1.7590 | 1.6833 | 1.8381 |  |
| Full sibling | 0.5891 | 0.0112 | <2.2*10^-308^ | 1.8023 | 1.7632 | 1.8423 | 0.4938 | 0.0175 | 1.1*10^-175^ | 1.6386 | 1.5834 | 1.6956 |  |
| Maternal half-sibling | 0.3056 | 0.0185 | 2.0*10^-61^ | 1.3575 | 1.3092 | 1.4076 | 0.2243 | 0.0286 | 4.9*10^-15^ | 1.2515 | 1.1831 | 1.3237 |  |
| Paternal half-sibling | 0.2261 | 0.0182 | 2.0*10^-35^ | 1.2537 | 1.2097 | 1.2992 | 0.1657 | 0.0289 | 1.0*10^-8^ | 1.1802 | 1.1152 | 1.2491 |  |
| With **OCD** | | | | | | | | | | | | |  |
| Mother-offspring | 0.4713 | 0.0236 | 1.6*10^-88^ | 1.6021 | 1.5296 | 1.6780 | 0.4352 | 0.0489 | 5.2*10^-19^ | 1.5453 | 1.4042 | 1.7006 |  |
| Father-offspring | 0.4164 | 0.0250 | 3.5*10^-62^ | 1.5164 | 1.4439 | 1.5927 | 0.4984 | 0.0635 | 4.2*10^-15^ | 1.6461 | 1.4535 | 1.8642 |  |
| Full sibling | 0.4699 | 0.0213 | 1.1*10^-107^ | 1.5998 | 1.5343 | 1.6680 | 0.3201 | 0.0334 | 1.0*10^-21^ | 1.3772 | 1.2899 | 1.4704 |  |
| Maternal half-sibling | 0.2205 | 0.0378 | 5.2*10^-9^ | 1.2467 | 1.1578 | 1.3425 | 0.1050 | 0.0611 | 8.5*10^-2^ | 1.1108 | 0.9855 | 1.2520 |  |
| Paternal half-sibling | 0.1922 | 0.0360 | 9.1*10^-8^ | 1.2119 | 1.1294 | 1.3004 | 0.0945 | 0.0618 | 1.3*10^-1^ | 1.0992 | 0.9737 | 1.2408 |  |
| With **PTSD** | | | | | | | | | | | | |  |
| Mother-offspring | 0.7801 | 0.0094 | <2.2*10^-308^ | 2.1817 | 2.1417 | 2.2224 | 0.7269 | 0.0131 | <2.2*10^-308^ | 2.0687 | 2.0161 | 2.1226 |  |
| Father-offspring | 0.6362 | 0.0104 | <2.2*10^-308^ | 1.8894 | 1.8511 | 1.9284 | 0.6337 | 0.0169 | <2.2*10^-308^ | 1.8845 | 1.8233 | 1.9479 |  |
| Full sibling | 0.6794 | 0.0095 | <2.2*10^-308^ | 1.9728 | 1.9365 | 2.0097 | 0.6356 | 0.0147 | <2.2*10^-308^ | 1.8881 | 1.8346 | 1.9433 |  |
| Maternal half-sibling | 0.3791 | 0.0154 | 1.5*10^-134^ | 1.4609 | 1.4176 | 1.5055 | 0.3143 | 0.0235 | 1.1*10^-40^ | 1.3693 | 1.3076 | 1.4339 |  |
| Paternal half-sibling | 0.2855 | 0.0152 | 2.2*10^-78^ | 1.3304 | 1.2913 | 1.3707 | 0.2214 | 0.0237 | 1.0*10^-20^ | 1.2478 | 1.1911 | 1.3072 |  |
| With **eating disorder** | | | | | | | | | | | | |  |
| Mother-offspring | 0.3431 | 0.0189 | 6.9*10^-74^ | 1.4093 | 1.3581 | 1.4624 | 0.3569 | 0.0368 | 3.4*10^-22^ | 1.4289 | 1.3293 | 1.5358 |  |
| Father-offspring | 0.2299 | 0.0203 | 9.9*10^-30^ | 1.2584 | 1.2094 | 1.3095 | 0.1299 | 0.0534 | 1.5*10^-2^ | 1.1387 | 1.0256 | 1.2644 |  |
| Full sibling | 0.3280 | 0.0171 | 4.4*10^-82^ | 1.3881 | 1.3424 | 1.4354 | 0.2289 | 0.0240 | 1.5*10^-21^ | 1.2572 | 1.1994 | 1.3177 |  |
| Maternal half-sibling | 0.1879 | 0.0297 | 2.4*10^-10^ | 1.2067 | 1.1385 | 1.2790 | 0.1361 | 0.0434 | 1.7*10^-3^ | 1.1458 | 1.0524 | 1.2475 |  |
| Paternal half-sibling | 0.1288 | 0.0287 | 7.2*10^-6^ | 1.1374 | 1.0752 | 1.2032 | 0.0429 | 0.0443 | 3.3*10^-1^ | 1.0439 | 0.9571 | 1.1385 |  |
| With **schizophrenia** | | | | | | | | | | | | | |
| Mother-offspring | 0.8002 | 0.0326 | 2.3*10^-133^ | 2.2260 | 2.0884 | 2.3727 | 0.6323 | 0.0429 | 2.9*10^-49^ | 1.8819 | 1.7303 | 2.0468 |  |
| Father-offspring | 0.6288 | 0.0353 | 7.0*10^-71^ | 1.8754 | 1.7499 | 2.0098 | 0.7589 | 0.0499 | 2.7*10^-52^ | 2.1360 | 1.9371 | 2.3554 |  |
| Full sibling | 0.6530 | 0.0299 | 1.1*10^-105^ | 1.9214 | 1.8120 | 2.0374 | 0.5550 | 0.0396 | 1.1*10^-44^ | 1.7420 | 1.6119 | 1.8825 |  |
| Maternal half-sibling | 0.3603 | 0.0540 | 2.5*10^-11^ | 1.4337 | 1.2898 | 1.5938 | 0.3103 | 0.0691 | 7.0*10^-6^ | 1.3638 | 1.1911 | 1.5615 |  |
| Paternal half-sibling | 0.2995 | 0.0555 | 6.7*10^-8^ | 1.3492 | 1.2102 | 1.5042 | 0.1918 | 0.0702 | 6.3*10^-3^ | 1.2114 | 1.0556 | 1.3902 |  |
| With **bipolar disorder** | | | | | | | | | | | | | |
| Mother-offspring | 0.8174 | 0.0167 | <2.2*10^-308^ | 2.2647 | 2.1917 | 2.3402 | 0.6124 | 0.0237 | 6.2*10^-147^ | 1.8449 | 1.7611 | 1.9327 |  |
| Father-offspring | 0.6832 | 0.0185 | 7.3*10^-299^ | 1.9802 | 1.9097 | 2.0533 | 0.5280 | 0.0300 | 1.7*10^-69^ | 1.6955 | 1.5988 | 1.7980 |  |
| Full sibling | 0.7098 | 0.0165 | <2.2*10^-308^ | 2.0336 | 1.9689 | 2.1005 | 0.5013 | 0.0278 | 1.3*10^-72^ | 1.6509 | 1.5633 | 1.7434 |  |
| Maternal half-sibling | 0.3504 | 0.0283 | 3.8*10^-35^ | 1.4196 | 1.3429 | 1.5006 | 0.2076 | 0.0490 | 2.3*10^-5^ | 1.2307 | 1.1180 | 1.3548 |  |
| Paternal half-sibling | 0.2866 | 0.0279 | 9.1*10^-25^ | 1.3319 | 1.2611 | 1.4068 | 0.2119 | 0.0479 | 9.8*10^-6^ | 1.2360 | 1.1252 | 1.3578 |  |
| With **other psychotic disorders (excl. SCZ)** | | | | | | | | | | | | | |
| Mother-offspring | 0.7993 | 0.0213 | 7.6*10^-307^ | 2.2239 | 2.1328 | 2.3189 | 0.5894 | 0.0259 | 1.2*10^-114^ | 1.8029 | 1.7137 | 1.8968 |  |
| Father-offspring | 0.6807 | 0.0227 | 6.3*10^-197^ | 1.9753 | 1.8892 | 2.0653 | 0.7074 | 0.0297 | 2.4*10^-125^ | 2.0287 | 1.9139 | 2.1503 |  |
| Full sibling | 0.6912 | 0.0197 | 1.6*10^-270^ | 1.9962 | 1.9207 | 2.0746 | 0.5819 | 0.0284 | 4.2*10^-93^ | 1.7895 | 1.6925 | 1.8921 |  |
| Maternal half-sibling | 0.3980 | 0.0331 | 2.5*10^-33^ | 1.4889 | 1.3954 | 1.5886 | 0.3561 | 0.0468 | 2.6*10^-14^ | 1.4278 | 1.3027 | 1.5649 |  |
| Paternal half-sibling | 0.3301 | 0.0335 | 7.2*10^-23^ | 1.3911 | 1.3026 | 1.4856 | 0.2051 | 0.0474 | 1.5*10^-5^ | 1.2277 | 1.1188 | 1.3472 |  |
| With **autism spectrum disorder** | | | | | | | | | | | | |  |
| Mother-offspring | 0.6686 | 0.0203 | 1.4*10^-237^ | 1.9515 | 1.8753 | 2.0307 | 0.6222 | 0.0542 | 1.9*10^-30^ | 1.8630 | 1.6751 | 2.0720 |  |
| Father-offspring | 0.4750 | 0.0218 | 5.9*10^-105^ | 1.6080 | 1.5407 | 1.6783 | 0.4513 | 0.0653 | 4.9*10^-12^ | 1.5703 | 1.3816 | 1.7848 |  |
| Full sibling | 0.5344 | 0.0186 | 2.2*10^-182^ | 1.7064 | 1.6455 | 1.7696 | 0.3694 | 0.0280 | 9.1*10^-40^ | 1.4469 | 1.3697 | 1.5285 |  |
| Maternal half-sibling | 0.2950 | 0.0294 | 9.1*10^-24^ | 1.3432 | 1.2681 | 1.4227 | 0.1601 | 0.0438 | 2.6*10^-4^ | 1.1737 | 1.0771 | 1.2789 |  |
| Paternal half-sibling | 0.2311 | 0.0304 | 2.6*10^-14^ | 1.2600 | 1.1873 | 1.3373 | 0.2154 | 0.0446 | 1.3*10^-6^ | 1.2403 | 1.1366 | 1.3535 |  |
| With **ADHD** | | | | | | | | | | | | |  |
| Mother-offspring | 0.9032 | 0.0120 | <2.2*10^-308^ | 2.4675 | 2.4102 | 2.5261 | 0.7635 | 0.0275 | 6.6*10^-170^ | 2.1458 | 2.0332 | 2.2645 |  |
| Father-offspring | 0.7817 | 0.0125 | <2.2*10^-308^ | 2.1851 | 2.1321 | 2.2394 | 0.7668 | 0.0303 | 2.0*10^-141^ | 2.1528 | 2.0288 | 2.2845 |  |
| Full sibling | 0.7548 | 0.0118 | <2.2*10^-308^ | 2.1272 | 2.0786 | 2.1770 | 0.5458 | 0.0184 | 1.7*10^-192^ | 1.7261 | 1.6648 | 1.7896 |  |
| Maternal half-sibling | 0.3947 | 0.0176 | 3.1*10^-111^ | 1.4840 | 1.4336 | 1.5361 | 0.2670 | 0.0254 | 9.2*10^-26^ | 1.3061 | 1.2425 | 1.3729 |  |
| Paternal half-sibling | 0.3730 | 0.0179 | 3.8*10^-96^ | 1.4521 | 1.4019 | 1.5040 | 0.2938 | 0.0263 | 6.3*10^-29^ | 1.3416 | 1.2741 | 1.4126 |  |

## **Table S10: Sex-specific** f**amilial coaggregation with other psychiatric disorders**

- - - - 1. **Suicide attempt**

|  | **Male** |  |  |  |  |  | **Female** |  |  |  |  |  |  | **Cross-sex** |  |  |  |  |  |  |
| --- | --- | --- | --- | --- | --- | --- | --- | --- | --- | --- | --- | --- | --- | --- | --- | --- | --- | --- | --- | --- |
| **Relative types** | **Coeff.** | **SE** | **p-value** | **OR** | **95% CI of OR** | | **Relative types** | **Coeff.** | **SE** | **p-value** | **OR** | **95% CI of OR** | | **Relative types** | **Coeff.** | **SE** | **p-value** | **OR** | **95% CI of OR** | |
| With **substance use disorder** | | | | | | | | | | | | | | | | | | | | |
|  |  |  |  |  |  |  |  |  |  |  |  |  |  | Mother-son | 1.0476 | 0.0131 | <2.2*10^-308^ | 2.8509 | 2.7786 | 2.925 |
| Father-son | 0.9745 | 0.0133 | <2.2*10^-308^ | 2.6498 | 2.5816 | 2.7198 | Mother-daughter | 1.1106 | 0.0144 | <2.2*10^-308^ | 3.0361 | 2.9515 | 3.1232 | Father-daughter | 0.9328 | 0.0151 | <2.2*10^-308^ | 2.5415 | 2.4673 | 2.6179 |
| Male full sibling | 1.0775 | 0.0186 | <2.2*10^-308^ | 2.9372 | 2.8322 | 3.0460 | Female full sibling | 1.0982 | 0.0202 | <2.2*10^-308^ | 2.9989 | 2.8824 | 3.1201 | Cross-sex full sibling | 0.9577 | 0.0136 | <2.2*10^-308^ | 2.6057 | 2.5370 | 2.6763 |
| Male maternal half-sibling | 0.5642 | 0.0291 | 1.2*10^-83^ | 1.758 | 1.6605 | 1.8613 | Female maternal half-sibling | 0.5626 | 0.0312 | 9.9*10^-73^ | 1.7552 | 1.6511 | 1.8659 | Cross-sex maternal half-sibling | 0.4828 | 0.0212 | 2*10^-114^ | 1.6206 | 1.5545 | 1.6894 |
| Male paternal half-sibling | 0.4195 | 0.0289 | 9.9*10^-48^ | 1.5212 | 1.4374 | 1.6099 | Female paternal half-sibling | 0.4279 | 0.031 | 3*10^-43^ | 1.5341 | 1.4435 | 1.6303 | Cross-sex paternal half-sibling | 0.396 | 0.0217 | 1.8*10^-74^ | 1.4859 | 1.4240 | 1.5504 |
| With **major depressive disorder** | | | | | | | | | | | | | | | | | | | | |
|  |  |  |  |  |  |  |  |  |  |  |  |  |  | Mother-son | 0.8426 | 0.0134 | <2.2*10^-308^ | 2.3223 | 2.2623 | 2.3840 |
| Father-son | 0.7735 | 0.0144 | <2.2*10^-308^ | 2.1673 | 2.1069 | 2.2295 | Mother-daughter | 0.845 | 0.0117 | <2.2*10^-308^ | 2.3279 | 2.2751 | 2.3819 | Father-daughter | 0.6849 | 0.0128 | <2.2*10^-308^ | 1.9837 | 1.9345 | 2.0341 |
| Male full sibling | 0.8166 | 0.0193 | <2.2*10^-308^ | 2.2627 | 2.1787 | 2.3499 | Female full sibling | 0.8366 | 0.0157 | <2.2*10^-308^ | 2.3086 | 2.2388 | 2.3807 | Cross-sex full sibling | 0.705 | 0.0123 | <2.2*10^-308^ | 2.0238 | 1.9756 | 2.0733 |
| Male maternal half-sibling | 0.397 | 0.0325 | 2.2*10^-34^ | 1.4874 | 1.3957 | 1.5851 | Female maternal half-sibling | 0.3989 | 0.0254 | 1.3*10^-55^ | 1.4902 | 1.4179 | 1.5662 | Cross-sex maternal half-sibling | 0.3029 | 0.0201 | 2*10^-51^ | 1.3538 | 1.3016 | 1.4082 |
| Male paternal half-sibling | 0.3112 | 0.0326 | 1.4*10^-21^ | 1.365 | 1.2805 | 1.4551 | Female paternal half-sibling | 0.3444 | 0.0254 | 5.2*10^-42^ | 1.4111 | 1.3427 | 1.4830 | Cross-sex paternal half-sibling | 0.2481 | 0.0204 | 4.8*10^-34^ | 1.2816 | 1.2314 | 1.3339 |
| With **anxiety** | | | | | | | | | | | | | | | | | | | | |
|  |  |  |  |  |  |  |  |  |  |  |  |  |  | Mother-son | 0.7700 | 0.0192 | <2.2*10^-308^ | 2.1599 | 2.0800 | 2.2427 |
| Father-son | 0.7295 | 0.0208 | 1.6*10^-268^ | 2.0741 | 1.9911 | 2.1605 | Mother-daughter | 0.7471 | 0.016 | <2.2*10^-308^ | 2.1109 | 2.0459 | 2.178 | Father-daughter | 0.6335 | 0.0175 | 1.8*10^-288^ | 1.8842 | 1.8208 | 1.9498 |
| Male full sibling | 0.8292 | 0.0274 | 1.4*10^-200^ | 2.2914 | 2.1714 | 2.418 | Female full sibling | 0.7735 | 0.0207 | 2.3*10^-306^ | 2.1674 | 2.0813 | 2.257 | Cross-sex full sibling | 0.6623 | 0.0171 | <2.2*10^-308^ | 1.9392 | 1.8755 | 2.0051 |
| Male maternal half-sibling | 0.4419 | 0.0449 | 7.6*10^-23^ | 1.5556 | 1.4245 | 1.6987 | Female maternal half-sibling | 0.3903 | 0.0334 | 1.7*10^-31^ | 1.4774 | 1.3837 | 1.5775 | Cross-sex maternal half-sibling | 0.2655 | 0.0275 | 4.8*10^-22^ | 1.304 | 1.2356 | 1.3763 |
| Male paternal half-sibling | 0.2333 | 0.0468 | 6.3*10^-7^ | 1.2627 | 1.1520 | 1.3841 | Female paternal half-sibling | 0.2965 | 0.0335 | 8.5*10^-19^ | 1.3451 | 1.2597 | 1.4364 | Cross-sex paternal half-sibling | 0.2468 | 0.0274 | 1.9*10^-19^ | 1.2799 | 1.2131 | 1.3505 |
| With **OCD** | | | | | | | | | | | | | | | | | | | | |
|  |  |  |  |  |  |  |  |  |  |  |  |  |  | Mother-son | 0.4621 | 0.0408 | 9.1*10^-30^ | 1.5875 | 1.4655 | 1.7196 |
| Father-son | 0.4974 | 0.0427 | 2.7*10^-31^ | 1.6444 | 1.5123 | 1.7882 | Mother-daughter | 0.6027 | 0.0323 | 1.1*10^-77^ | 1.8270 | 1.7149 | 1.9464 | Father-daughter | 0.5553 | 0.0347 | 1.6*10^-57^ | 1.7424 | 1.6278 | 1.8652 |
| Male full sibling | 0.4606 | 0.0578 | 1.6*10^-15^ | 1.585 | 1.4152 | 1.7753 | Female full sibling | 0.7122 | 0.0396 | 3.7*10^-72^ | 2.0385 | 1.8861 | 2.2032 | Cross-sex full sibling | 0.5427 | 0.0332 | 4.1*10^-60^ | 1.7207 | 1.6124 | 1.8364 |
| Male maternal half-sibling | 0.1818 | 0.1007 | 7.1*10^-2^ | 1.1993 | 0.9846 | 1.4609 | Female maternal half-sibling | 0.2928 | 0.067 | 1.2*10^-5^ | 1.3401 | 1.1752 | 1.5282 | Cross-sex maternal half-sibling | 0.1667 | 0.0567 | 3.3*10^-3^ | 1.1813 | 1.0571 | 1.3202 |
| Male paternal half-sibling | 0.0622 | 0.0965 | 5.2*10^-1^ | 1.0642 | 0.8808 | 1.2857 | Female paternal half-sibling | 0.2638 | 0.0651 | 5*10^-5^ | 1.3018 | 1.1459 | 1.4789 | Cross-sex paternal half-sibling | 0.2592 | 0.0531 | 1*10^-6^ | 1.2958 | 1.1678 | 1.4379 |
| With **PTSD** | | | | | | | | | | | | | | | | | | | | |
|  |  |  |  |  |  |  |  |  |  |  |  |  |  | Mother-son | 0.9410 | 0.0155 | <2.2*10^-308^ | 2.5626 | 2.4858 | 2.6418 |
| Father-son | 0.8403 | 0.0173 | <2.2*10^-308^ | 2.3171 | 2.2397 | 2.3972 | Mother-daughter | 0.9102 | 0.0128 | <2.2*10^-308^ | 2.4849 | 2.4235 | 2.5479 | Father-daughter | 0.7325 | 0.0142 | <2.2*10^-308^ | 2.0804 | 2.0233 | 2.139 |
| Male full sibling | 0.8773 | 0.0233 | <2.2*10^-308^ | 2.4044 | 2.297 | 2.5168 | Female full sibling | 0.8772 | 0.0175 | <2.2*10^-308^ | 2.4042 | 2.3232 | 2.4881 | Cross-sex full sibling | 0.7510 | 0.0141 | <2.2*10^-308^ | 2.1191 | 2.0612 | 2.1787 |
| Male maternal half-sibling | 0.4443 | 0.0383 | 3.7*10^-31^ | 1.5594 | 1.4467 | 1.6809 | Female maternal half-sibling | 0.4523 | 0.0282 | 4.8*10^-58^ | 1.5720 | 1.4876 | 1.6612 | Cross-sex maternal half-sibling | 0.3856 | 0.0227 | 7.2*10^-65^ | 1.4705 | 1.4066 | 1.5373 |
| Male paternal half-sibling | 0.3497 | 0.0373 | 7.5*10^-21^ | 1.4187 | 1.3185 | 1.5264 | Female paternal half-sibling | 0.3622 | 0.0277 | 3.6*10^-39^ | 1.4365 | 1.3607 | 1.5166 | Cross-sex paternal half-sibling | 0.2596 | 0.0232 | 4.8*10^-29^ | 1.2965 | 1.2388 | 1.3568 |
| With **eating disorder** | | | | | | | | | | | | | | | | | | | | |
|  |  |  |  |  |  |  |  |  |  |  |  |  |  | Mother-son | 0.4971 | 0.0416 | 7.7*10^-33^ | 1.6439 | 1.5151 | 1.7837 |
| Father-son | 0.2706 | 0.0509 | 1.1*10^-7^ | 1.3107 | 1.1862 | 1.4482 | Mother-daughter | 0.3793 | 0.0234 | 7.1*10^-59^ | 1.4613 | 1.3957 | 1.5300 | Father-daughter | 0.3129 | 0.0250 | 6.2*10^-36^ | 1.3675 | 1.3020 | 1.4361 |
| Male full sibling | 0.4198 | 0.0643 | 6.4*10^-11^ | 1.5216 | 1.3416 | 1.7258 | Female full sibling | 0.4889 | 0.0283 | 8.7*10^-67^ | 1.6306 | 1.5425 | 1.7236 | Cross-sex full sibling | 0.2635 | 0.0281 | 6.8*10^-21^ | 1.3014 | 1.2317 | 1.3751 |
| Male maternal half-sibling | 0.1952 | 0.1043 | 6.1*10^-2^ | 1.2156 | 0.9908 | 1.4914 | Female maternal half-sibling | 0.2662 | 0.0478 | 2.5*10^-8^ | 1.3050 | 1.1884 | 1.4330 | Cross-sex maternal half-sibling | 0.0526 | 0.0470 | 2.6*10^-1^ | 1.0540 | 0.9612 | 1.1557 |
| Male paternal half-sibling | 0.1270 | 0.1009 | 2.1*10^-1^ | 1.1355 | 0.9317 | 1.3838 | Female paternal half-sibling | 0.193 | 0.0472 | 4.3*10^-5^ | 1.2128 | 1.1057 | 1.3303 | Cross-sex paternal half-sibling | -0.0008 | 0.0465 | 9.9*10^-1^ | 0.9992 | 0.9123 | 1.0945 |
| With **schizophrenia** | | | | | | | | | | | | | | | | | | | | |
|  |  |  |  |  |  |  |  |  |  |  |  |  |  | Mother-son | 0.9937 | 0.0435 | 1.9*10^-115^ | 2.7013 | 2.4805 | 2.9418 |
| Father-son | 0.6963 | 0.0491 | 1.4*10^-45^ | 2.0063 | 1.8221 | 2.2091 | Mother-daughter | 0.8903 | 0.0541 | 7.2*10^-61^ | 2.436 | 2.1909 | 2.7084 | Father-daughter | 0.7674 | 0.0573 | 6.4*10^-41^ | 2.1542 | 1.9254 | 2.4102 |
| Male full sibling | 0.8703 | 0.0567 | 4*10^-53^ | 2.3876 | 2.1364 | 2.6684 | Female full sibling | 0.8908 | 0.0664 | 4.5*10^-41^ | 2.4371 | 2.1398 | 2.7757 | Cross-sex full sibling | 0.7897 | 0.0427 | 2.2*10^-76^ | 2.2028 | 2.026 | 2.3951 |
| Male maternal half-sibling | 0.4195 | 0.1038 | 5.3*10^-5^ | 1.5213 | 1.2413 | 1.8644 | Female maternal half-sibling | 0.5633 | 0.1136 | 7*10^-7^ | 1.7565 | 1.4060 | 2.1944 | Cross-sex maternal half-sibling | 0.4408 | 0.0758 | 6.2*10^-9^ | 1.5539 | 1.3393 | 1.8029 |
| Male paternal half-sibling | 0.2952 | 0.1002 | 3.2*10^-3^ | 1.3434 | 1.1038 | 1.635 | Female paternal half-sibling | 0.328 | 0.1224 | 7.4*10^-3^ | 1.3882 | 1.0920 | 1.7647 | Cross-sex paternal half-sibling | 0.3748 | 0.079 | 2.1*10^-6^ | 1.4547 | 1.2461 | 1.6982 |
| With **bipolar disorder** | | | | | | | | | | | | | | | | | | | | |
|  |  |  |  |  |  |  |  |  |  |  |  |  |  | Mother-son | 0.9096 | 0.0286 | 1.6*10^-222^ | 2.4834 | 2.3482 | 2.6265 |
| Father-son | 0.8746 | 0.0311 | 1.7*10^-173^ | 2.3978 | 2.2558 | 2.5488 | Mother-daughter | 0.9519 | 0.0224 | <2.2*10^-308^ | 2.5906 | 2.4795 | 2.7068 | Father-daughter | 0.7628 | 0.0252 | 1.2*10^-201^ | 2.1443 | 2.0410 | 2.2528 |
| Male full sibling | 0.7780 | 0.0432 | 2.3*10^-72^ | 2.1772 | 2.0002 | 2.3698 | Female full sibling | 0.9424 | 0.0299 | 1.6*10^-218^ | 2.5661 | 2.4202 | 2.7208 | Cross-sex full sibling | 0.7938 | 0.0250 | 4.1*10^-221^ | 2.2117 | 2.1059 | 2.3228 |
| Male maternal half-sibling | 0.3775 | 0.0734 | 2.7*10^-7^ | 1.4586 | 1.2632 | 1.6843 | Female maternal half-sibling | 0.5030 | 0.0485 | 3.4*10^-25^ | 1.6537 | 1.5037 | 1.8186 | Cross-sex maternal half-sibling | 0.2847 | 0.0421 | 1.3*10^-11^ | 1.3294 | 1.2242 | 1.4437 |
| Male paternal half-sibling | 0.2747 | 0.0747 | 2.3*10^-4^ | 1.3161 | 1.1369 | 1.5235 | Female paternal half-sibling | 0.3974 | 0.0481 | 1.5*10^-16^ | 1.4879 | 1.3540 | 1.6351 | Cross-sex paternal half-sibling | 0.2696 | 0.0425 | 2.2*10^-10^ | 1.3094 | 1.2048 | 1.4231 |
| With **other psychotic disorders (excl. SCZ)** | | | | | | | | | | | | | | | | | | | | |
|  |  |  |  |  |  |  |  |  |  |  |  |  |  | Mother-son | 0.9419 | 0.0294 | 2.5*10^-225^ | 2.5649 | 2.4213 | 2.7170 |
| Father-son | 0.8098 | 0.0316 | 1.6*10^-144^ | 2.2474 | 2.1123 | 2.3912 | Mother-daughter | 0.9100 | 0.0343 | 2*10^-155^ | 2.4842 | 2.3229 | 2.6568 | Father-daughter | 0.7696 | 0.0372 | 3.9*10^-95^ | 2.1590 | 2.0072 | 2.3222 |
| Male full sibling | 0.9368 | 0.0396 | 1.1*10^-123^ | 2.5518 | 2.3612 | 2.7577 | Female full sibling | 0.8810 | 0.0432 | 2.3*10^-92^ | 2.4133 | 2.2173 | 2.6266 | Cross-sex full sibling | 0.8130 | 0.0293 | 1*10^-169^ | 2.2547 | 2.1289 | 2.3879 |
| Male maternal half-sibling | 0.4926 | 0.0659 | 7.6*10^-14^ | 1.6366 | 1.4383 | 1.8622 | Female maternal half-sibling | 0.5210 | 0.0747 | 3*10^-12^ | 1.6837 | 1.4544 | 1.9491 | Cross-sex maternal half-sibling | 0.4552 | 0.0476 | 1.1*10^-21^ | 1.5765 | 1.4361 | 1.7306 |
| Male paternal half-sibling | 0.4229 | 0.0634 | 2.6*10^-11^ | 1.5264 | 1.3480 | 1.7285 | Female paternal half-sibling | 0.3804 | 0.0720 | 1.3*10^-7^ | 1.4629 | 1.2702 | 1.6847 | Cross-sex paternal half-sibling | 0.3918 | 0.0489 | 1.1*10^-15^ | 1.4797 | 1.3445 | 1.6284 |
| With **autism spectrum disorder** | | | | | | | | | | | | | | | | | | | | |
|  |  |  |  |  |  |  |  |  |  |  |  |  |  | Mother-son | 0.7492 | 0.0274 | 6.2*10^-165^ | 2.1153 | 2.0048 | 2.2318 |
| Father-son | 0.6317 | 0.0296 | 6.4*10^-101^ | 1.8808 | 1.7747 | 1.9932 | Mother-daughter | 0.7774 | 0.0333 | 1.3*10^-120^ | 2.1758 | 2.0383 | 2.3225 | Father-daughter | 0.5388 | 0.0380 | 1.5*10^-45^ | 1.7140 | 1.5909 | 1.8467 |
| Male full sibling | 0.6929 | 0.0392 | 5.3*10^-70^ | 1.9995 | 1.8517 | 2.1591 | Female full sibling | 0.7889 | 0.0418 | 1.8*10^-79^ | 2.2010 | 2.0279 | 2.3889 | Cross-sex full sibling | 0.7166 | 0.0279 | 2.2*10^-145^ | 2.0475 | 1.9385 | 2.1626 |
| Male maternal half-sibling | 0.2832 | 0.0618 | 4.6*10^-6^ | 1.3273 | 1.1759 | 1.4983 | Female maternal half-sibling | 0.4320 | 0.0645 | 2.1*10^-11^ | 1.5404 | 1.3574 | 1.7480 | Cross-sex maternal half-sibling | 0.4119 | 0.0421 | 1.3*10^-22^ | 1.5097 | 1.3902 | 1.6395 |
| Male paternal half-sibling | 0.3248 | 0.0608 | 9.4*10^-8^ | 1.3838 | 1.2282 | 1.5590 | Female paternal half-sibling | 0.4006 | 0.0650 | 7*10^-10^ | 1.4927 | 1.3142 | 1.6954 | Cross-sex paternal half-sibling | 0.2858 | 0.0446 | 1.5*10^-10^ | 1.3308 | 1.2194 | 1.4525 |
| With **ADHD** | | | | | | | | | | | | | | | | | | | | |
|  |  |  |  |  |  |  |  |  |  |  |  |  |  | Mother-son | 1.0233 | 0.0166 | <2.2*10^-308^ | 2.7825 | 2.6937 | 2.8743 |
| Father-son | 0.9592 | 0.0175 | <2.2*10^-308^ | 2.6097 | 2.5215 | 2.7009 | Mother-daughter | 1.0289 | 0.0181 | <2.2*10^-308^ | 2.7981 | 2.7006 | 2.8992 | Father-daughter | 0.8927 | 0.0197 | <2.2*10^-308^ | 2.4417 | 2.3491 | 2.5379 |
| Male full sibling | 0.9750 | 0.0255 | <2.2*10^-308^ | 2.6512 | 2.5221 | 2.7868 | Female full sibling | 0.9523 | 0.0254 | 1*10^-307^ | 2.5916 | 2.4657 | 2.7238 | Cross-sex full sibling | 0.9186 | 0.0174 | <2.2*10^-308^ | 2.5059 | 2.4220 | 2.5927 |
| Male maternal half-sibling | 0.4948 | 0.0367 | 1.6*10^-41^ | 1.6401 | 1.5265 | 1.7623 | Female maternal half-sibling | 0.4807 | 0.0356 | 1.3*10^-41^ | 1.6171 | 1.5083 | 1.7339 | Cross-sex maternal half-sibling | 0.4344 | 0.0254 | 1.8*10^-65^ | 1.5440 | 1.4690 | 1.6229 |
| Male paternal half-sibling | 0.4771 | 0.0364 | 3.6*10^-39^ | 1.6115 | 1.5004 | 1.7308 | Female paternal half-sibling | 0.4514 | 0.0362 | 1.1*10^-35^ | 1.5706 | 1.4630 | 1.6861 | Cross-sex paternal half-sibling | 0.3952 | 0.0263 | 6.6*10^-51^ | 1.4847 | 1.4100 | 1.5634 |

- - - - 1. **Self-harm**

|  | **Male** |  |  |  |  |  | **Female** |  |  |  |  |  |  | **Cross-sex** |  |  |  |  |  |  |
| --- | --- | --- | --- | --- | --- | --- | --- | --- | --- | --- | --- | --- | --- | --- | --- | --- | --- | --- | --- | --- |
| **Relative types** | **Coeff.** | **SE** | **p-value** | **OR** | **95% CI of OR** |  | **Relative types** | **Coeff.** | **SE** | **p-value** | **OR** | **95% CI of OR** |  | **Relative types** | **Coeff.** | **SE** | **p-value** | **OR** | **95% CI of OR** |  |
| With **substance use disorder** | | | | | | | | | | | | | | | | | | | | |
|  |  |  |  |  |  |  |  |  |  |  |  |  |  | Mother-son | 0.8909 | 0.0123 | <2.2*10^-308^ | 2.4374 | 2.3795 | 2.4966 |
| Father-son | 0.8094 | 0.0123 | <2.2*10^-308^ | 2.2465 | 2.1931 | 2.3012 | Mother-daughter | 0.9696 | 0.0136 | <2.2*10^-308^ | 2.6369 | 2.5675 | 2.7081 | Father-daughter | 0.7857 | 0.0141 | <2.2*10^-308^ | 2.1939 | 2.1340 | 2.2554 |
| Male full sibling | 0.8228 | 0.0163 | <2.2*10^-308^ | 2.2769 | 2.2054 | 2.3507 | Female full sibling | 0.9696 | 0.0188 | <2.2*10^-308^ | 2.6369 | 2.5413 | 2.7361 | Cross-sex full sibling | 0.7797 | 0.0123 | <2.2*10^-308^ | 2.1808 | 2.1287 | 2.2342 |
| Male maternal half-sibling | 0.4813 | 0.0258 | 1.2*10^-77^ | 1.6182 | 1.5384 | 1.7021 | Female maternal half-sibling | 0.5258 | 0.0293 | 7.6*10^-72^ | 1.6917 | 1.5972 | 1.7918 | Cross-sex maternal half-sibling | 0.4171 | 0.0196 | 1.9*10^-100^ | 1.5175 | 1.4603 | 1.5769 |
| Male paternal half-sibling | 0.3558 | 0.0257 | 2*10^-43^ | 1.4273 | 1.3570 | 1.5011 | Female paternal half-sibling | 0.3974 | 0.0291 | 1.4*10^-42^ | 1.4879 | 1.4056 | 1.5751 | Cross-sex paternal half-sibling | 0.3279 | 0.0198 | 2*10^-61^ | 1.3880 | 1.3351 | 1.4430 |
| With **major depressive disorder** | | | | | | | | | | | | | | | | | | | | |
|  |  |  |  |  |  |  |  |  |  |  |  |  |  | Mother-son | 0.7052 | 0.0123 | <2.2*10^-308^ | 2.0242 | 1.9759 | 2.0736 |
| Father-son | 0.6281 | 0.0133 | <2.2*10^-308^ | 1.8740 | 1.8258 | 1.9234 | Mother-daughter | 0.7208 | 0.0109 | <2.2*10^-308^ | 2.0561 | 2.0127 | 2.1005 | Father-daughter | 0.5593 | 0.0118 | <2.2*10^-308^ | 1.7494 | 1.7094 | 1.7903 |
| Male full sibling | 0.5970 | 0.0169 | 1.3*10^-274^ | 1.8166 | 1.7576 | 1.8776 | Female full sibling | 0.7207 | 0.0144 | <2.2*10^-308^ | 2.0558 | 1.9986 | 2.1147 | Cross-sex full sibling | 0.5720 | 0.0109 | <2.2*10^-308^ | 1.7717 | 1.7341 | 1.8101 |
| Male maternal half-sibling | 0.3149 | 0.0288 | 7.3*10^-28^ | 1.3701 | 1.2949 | 1.4496 | Female maternal half-sibling | 0.3645 | 0.0239 | 1.6*10^-52^ | 1.4398 | 1.3739 | 1.5089 | Cross-sex maternal half-sibling | 0.2595 | 0.0184 | 5.3*10^-45^ | 1.2962 | 1.2503 | 1.3439 |
| Male paternal half-sibling | 0.2491 | 0.0292 | 1.5*10^-17^ | 1.2829 | 1.2115 | 1.3585 | Female paternal half-sibling | 0.2937 | 0.0238 | 5.6*10^-35^ | 1.3414 | 1.2803 | 1.4055 | Cross-sex paternal half-sibling | 0.2173 | 0.0186 | 1.2*10^-31^ | 1.2427 | 1.1983 | 1.2888 |
| With **anxiety** | | | | | | | | | | | | | | | | | | | | |
|  |  |  |  |  |  |  |  |  |  |  |  |  |  | Mother-son | 0.6482 | 0.0178 | 9.7*10^-292^ | 1.912 | 1.8466 | 1.9797 |
| Father-son | 0.5963 | 0.0192 | 6.1*10^-212^ | 1.8153 | 1.7483 | 1.8849 | Mother-daughter | 0.6396 | 0.0150 | <2.2*10^-308^ | 1.8958 | 1.8410 | 1.9522 | Father-daughter | 0.5191 | 0.0162 | 5.6*10^-226^ | 1.6806 | 1.6281 | 1.7347 |
| Male full sibling | 0.6022 | 0.0244 | 7.1*10^-135^ | 1.8262 | 1.7410 | 1.9155 | Female full sibling | 0.6593 | 0.0193 | 6.3*10^-255^ | 1.9335 | 1.8616 | 2.0082 | Cross-sex full sibling | 0.5327 | 0.0153 | 3.3*10^-265^ | 1.7035 | 1.6531 | 1.7554 |
| Male maternal half-sibling | 0.3124 | 0.0408 | 2*10^-14^ | 1.3667 | 1.2616 | 1.4805 | Female maternal half-sibling | 0.3591 | 0.0319 | 2.2*10^-29^ | 1.432 | 1.3452 | 1.5244 | Cross-sex maternal half-sibling | 0.2475 | 0.0252 | 7.7*10^-23^ | 1.2808 | 1.2192 | 1.3456 |
| Male paternal half-sibling | 0.1785 | 0.0422 | 2.3*10^-5^ | 1.1955 | 1.1006 | 1.2985 | Female paternal half-sibling | 0.2474 | 0.0317 | 6.2*10^-15^ | 1.2807 | 1.2035 | 1.3629 | Cross-sex paternal half-sibling | 0.2113 | 0.0249 | 2*10^-17^ | 1.2353 | 1.1765 | 1.2971 |
| With **OCD** | | | | | | | | | | | | | | | | | | | | |
|  |  |  |  |  |  |  |  |  |  |  |  |  |  | Mother-son | 0.3882 | 0.0375 | 4.6*10^-25^ | 1.4743 | 1.3697 | 1.5868 |
| Father-son | 0.3921 | 0.0392 | 1.5*10^-23^ | 1.4801 | 1.3706 | 1.5983 | Mother-daughter | 0.5201 | 0.0302 | 1.6*10^-66^ | 1.6823 | 1.5856 | 1.7848 | Father-daughter | 0.4357 | 0.0322 | 7.9*10^-42^ | 1.5461 | 1.4516 | 1.6466 |
| Male full sibling | 0.2899 | 0.0494 | 4.4*10^-9^ | 1.3363 | 1.2130 | 1.4722 | Female full sibling | 0.6095 | 0.0371 | 9.6*10^-61^ | 1.8396 | 1.7107 | 1.9782 | Cross-sex full sibling | 0.4458 | 0.0293 | 3.7*10^-52^ | 1.5618 | 1.4745 | 1.6542 |
| Male maternal half-sibling | 0.1079 | 0.0897 | 2.3*10^-1^ | 1.1140 | 0.9343 | 1.3282 | Female maternal half-sibling | 0.2875 | 0.0630 | 5*10^-6^ | 1.3331 | 1.1783 | 1.5084 | Cross-sex maternal half-sibling | 0.1948 | 0.0508 | 1.3*10^-4^ | 1.2151 | 1.0998 | 1.3424 |
| Male paternal half-sibling | 0.0086 | 0.0876 | 9.2*10^-1^ | 1.0086 | 0.8494 | 1.1976 | Female paternal half-sibling | 0.2584 | 0.0612 | 2.4*10^-5^ | 1.2949 | 1.1484 | 1.4600 | Cross-sex paternal half-sibling | 0.1993 | 0.0493 | 5.2*10^-5^ | 1.2206 | 1.1083 | 1.3443 |
| With **PTSD** | | | | | | | | | | | | | | | | | | | | |
|  |  |  |  |  |  |  |  |  |  |  |  |  |  | Mother-son | 0.7668 | 0.0144 | <2.2*10^-308^ | 2.1529 | 2.0930 | 2.2145 |
| Father-son | 0.6924 | 0.0160 | <2.2*10^-308^ | 1.9985 | 1.9367 | 2.0622 | Mother-daughter | 0.7841 | 0.0120 | <2.2*10^-308^ | 2.1904 | 2.1397 | 2.2424 | Father-daughter | 0.6081 | 0.0132 | <2.2*10^-308^ | 1.837 | 1.7902 | 1.8850 |
| Male full sibling | 0.6785 | 0.0205 | 3*10^-239^ | 1.9708 | 1.8931 | 2.0518 | Female full sibling | 0.7620 | 0.0162 | <2.2*10^-308^ | 2.1425 | 2.0753 | 2.2117 | Cross-sex full sibling | 0.6195 | 0.0127 | <2.2*10^-308^ | 1.8579 | 1.8124 | 1.9046 |
| Male maternal half-sibling | 0.3561 | 0.0340 | 1.2*10^-25^ | 1.4278 | 1.3357 | 1.5263 | Female maternal half-sibling | 0.4194 | 0.0267 | 1.9*10^-55^ | 1.5210 | 1.4433 | 1.6028 | Cross-sex maternal half-sibling | 0.3356 | 0.0209 | 6.6*10^-58^ | 1.3988 | 1.3426 | 1.4574 |
| Male paternal half-sibling | 0.2724 | 0.0336 | 5*10^-16^ | 1.3131 | 1.2295 | 1.4024 | Female paternal half-sibling | 0.3292 | 0.0261 | 1.6*10^-36^ | 1.3898 | 1.3206 | 1.4627 | Cross-sex paternal half-sibling | 0.2395 | 0.0209 | 2.2*10^-30^ | 1.2706 | 1.2196 | 1.3238 |
| With **eating disorder** | | | | | | | | | | | | | | | | | | | | |
|  |  |  |  |  |  |  |  |  |  |  |  |  |  | Mother-son | 0.4009 | 0.0381 | 6.2*10^-26^ | 1.4931 | 1.3858 | 1.6087 |
| Father-son | 0.2122 | 0.0459 | 3.7*10^-6^ | 1.2364 | 1.1302 | 1.3527 | Mother-daughter | 0.3196 | 0.0216 | 1.9*10^-49^ | 1.3765 | 1.3194 | 1.4361 | Father-daughter | 0.2477 | 0.0225 | 4.4*10^-28^ | 1.2811 | 1.2257 | 1.3390 |
| Male full sibling | 0.2507 | 0.0561 | 7.9*10^-6^ | 1.2849 | 1.1511 | 1.4343 | Female full sibling | 0.4182 | 0.0258 | 2.6*10^-59^ | 1.5193 | 1.4445 | 1.5979 | Cross-sex full sibling | 0.2397 | 0.0240 | 1.6*10^-23^ | 1.2708 | 1.2125 | 1.3320 |
| Male maternal half-sibling | 0.0994 | 0.0948 | 2.9*10^-1^ | 1.1046 | 0.9173 | 1.3300 | Female maternal half-sibling | 0.2813 | 0.0446 | 2.9*10^-10^ | 1.3248 | 1.2139 | 1.4459 | Cross-sex maternal half-sibling | 0.0692 | 0.0420 | 9.9*10^-2^ | 1.0717 | 0.9870 | 1.1636 |
| Male paternal half-sibling | 0.1965 | 0.0846 | 2*10^-2^ | 1.2171 | 1.0311 | 1.4368 | Female paternal half-sibling | 0.1490 | 0.0444 | 7.9*10^-4^ | 1.1606 | 1.0639 | 1.2662 | Cross-sex paternal half-sibling | 0.0521 | 0.0411 | 2.1*10^-1^ | 1.0534 | 0.9719 | 1.1419 |
| With **schizophrenia** | | | | | | | | | | | | | | | | | | | | |
|  |  |  |  |  |  |  |  |  |  |  |  |  |  | Mother-son | 0.8369 | 0.0413 | 2.3*10^-91^ | 2.3092 | 2.1297 | 2.5038 |
| Father-son | 0.6174 | 0.0455 | 5.4*10^-42^ | 1.8541 | 1.6960 | 2.0269 | Mother-daughter | 0.7503 | 0.0516 | 6*10^-48^ | 2.1176 | 1.9140 | 2.3428 | Father-daughter | 0.6416 | 0.0547 | 9.3*10^-32^ | 1.8995 | 1.7064 | 2.1145 |
| Male full sibling | 0.6383 | 0.0525 | 5.7*10^-34^ | 1.8933 | 1.7081 | 2.0987 | Female full sibling | 0.7746 | 0.0637 | 4.7*10^-34^ | 2.1696 | 1.9151 | 2.4580 | Cross-sex full sibling | 0.6149 | 0.0400 | 2.3*10^-53^ | 1.8495 | 1.7100 | 2.0002 |
| Male maternal half-sibling | 0.2759 | 0.0969 | 4.4*10^-3^ | 1.3178 | 1.0899 | 1.5933 | Female maternal half-sibling | 0.4973 | 0.1088 | 4.9*10^-6^ | 1.6443 | 1.3285 | 2.0351 | Cross-sex maternal half-sibling | 0.3614 | 0.0721 | 5.3*10^-7^ | 1.4353 | 1.2462 | 1.6531 |
| Male paternal half-sibling | 0.2404 | 0.0932 | 9.9*10^-3^ | 1.2717 | 1.0594 | 1.5266 | Female paternal half-sibling | 0.3219 | 0.1140 | 4.8*10^-3^ | 1.3797 | 1.1033 | 1.7253 | Cross-sex paternal half-sibling | 0.3386 | 0.0731 | 3.7*10^-6^ | 1.4030 | 1.2156 | 1.6192 |
| With **bipolar disorder** | | | | | | | | | | | | | | | | | | | | |
|  |  |  |  |  |  |  |  |  |  |  |  |  |  | Mother-son | 0.7649 | 0.0264 | 4.9*10^-184^ | 2.1487 | 2.0402 | 2.2629 |
| Father-son | 0.7358 | 0.0290 | 4.4*10^-142^ | 2.0871 | 1.9718 | 2.2092 | Mother-daughter | 0.8457 | 0.0210 | <2.2*10^-308^ | 2.3295 | 2.2355 | 2.4274 | Father-daughter | 0.6567 | 0.0234 | 2.8*10^-173^ | 1.9284 | 1.8419 | 2.0189 |
| Male full sibling | 0.6159 | 0.0376 | 2*10^-60^ | 1.8513 | 1.7199 | 1.9928 | Female full sibling | 0.8095 | 0.0283 | 1.1*10^-179^ | 2.2469 | 2.1256 | 2.3751 | Cross-sex full sibling | 0.6727 | 0.0226 | 3.5*10^-195^ | 1.9595 | 1.8747 | 2.0482 |
| Male maternal half-sibling | 0.3250 | 0.0658 | 7.7*10^-7^ | 1.3840 | 1.2166 | 1.5744 | Female maternal half-sibling | 0.4633 | 0.0460 | 8*10^-24^ | 1.5892 | 1.4521 | 1.7393 | Cross-sex maternal half-sibling | 0.2520 | 0.0395 | 1.7*10^-10^ | 1.2865 | 1.1908 | 1.3900 |
| Male paternal half-sibling | 0.2121 | 0.0680 | 1.8*10^-3^ | 1.2363 | 1.0820 | 1.4127 | Female paternal half-sibling | 0.3536 | 0.0456 | 9.1*10^-15^ | 1.4243 | 1.3024 | 1.5575 | Cross-sex paternal half-sibling | 0.2436 | 0.0385 | 2.4*10^-10^ | 1.2759 | 1.1832 | 1.3757 |
| With **other psychotic disorders (excl. SCZ)** | | | | | | | | | | | | | | | | | | | | |
|  |  |  |  |  |  |  |  |  |  |  |  |  |  | Mother-son | 0.8037 | 0.0277 | 3.2*10^-185^ | 2.2338 | 2.1158 | 2.3584 |
| Father-son | 0.6983 | 0.0293 | 1.2*10^-125^ | 2.0103 | 1.8981 | 2.1290 | Mother-daughter | 0.7982 | 0.0323 | 7.9*10^-135^ | 2.2215 | 2.0852 | 2.3667 | Father-daughter | 0.6538 | 0.0349 | 2.1*10^-78^ | 1.9228 | 1.7957 | 2.0588 |
| Male full sibling | 0.7228 | 0.0354 | 7.1*10^-93^ | 2.0602 | 1.9223 | 2.2081 | Female full sibling | 0.7658 | 0.0409 | 2.4*10^-78^ | 2.1507 | 1.9852 | 2.3301 | Cross-sex full sibling | 0.6447 | 0.0269 | 1*10^-126^ | 1.9054 | 1.8075 | 2.0087 |
| Male maternal half-sibling | 0.3998 | 0.0590 | 1.3*10^-11^ | 1.4915 | 1.3286 | 1.6745 | Female maternal half-sibling | 0.4560 | 0.0722 | 2.7*10^-10^ | 1.5777 | 1.3696 | 1.8175 | Cross-sex maternal half-sibling | 0.3881 | 0.0444 | 2.2*10^-18^ | 1.4742 | 1.3514 | 1.6081 |
| Male paternal half-sibling | 0.3231 | 0.0588 | 3.9*10^-8^ | 1.3814 | 1.2310 | 1.5501 | Female paternal half-sibling | 0.3623 | 0.0671 | 6.7*10^-8^ | 1.4367 | 1.2596 | 1.6387 | Cross-sex paternal half-sibling | 0.3335 | 0.0456 | 2.7*10^-13^ | 1.3958 | 1.2764 | 1.5264 |
| With **autism spectrum disorder** | | | | | | | | | | | | | | | | | | | | |
|  |  |  |  |  |  |  |  |  |  |  |  |  |  | Mother-son | 0.6678 | 0.0256 | 3.2*10^-150^ | 1.9500 | 1.8546 | 2.0503 |
| Father-son | 0.5129 | 0.0271 | 4.3*10^-80^ | 1.6701 | 1.5839 | 1.7611 | Mother-daughter | 0.6814 | 0.0314 | 2.9*10^-104^ | 1.9767 | 1.8586 | 2.1023 | Father-daughter | 0.4111 | 0.0351 | 1.2*10^-31^ | 1.5084 | 1.4081 | 1.6159 |
| Male full sibling | 0.4342 | 0.0339 | 1.6*10^-37^ | 1.5437 | 1.4444 | 1.6497 | Female full sibling | 0.7008 | 0.0387 | 3*10^-73^ | 2.0153 | 1.8681 | 2.1742 | Cross-sex full sibling | 0.5338 | 0.0252 | 2.1*10^-99^ | 1.7054 | 1.6231 | 1.7918 |
| Male maternal half-sibling | 0.1686 | 0.0559 | 2.5*10^-3^ | 1.1837 | 1.0609 | 1.3207 | Female maternal half-sibling | 0.4192 | 0.0608 | 5.5*10^-12^ | 1.5208 | 1.3499 | 1.7133 | Cross-sex maternal half-sibling | 0.3319 | 0.0393 | 2.8*10^-17^ | 1.3936 | 1.2904 | 1.5051 |
| Male paternal half-sibling | 0.2322 | 0.0539 | 1.7*10^-5^ | 1.2613 | 1.1348 | 1.4019 | Female paternal half-sibling | 0.3216 | 0.0626 | 2.8*10^-7^ | 1.3793 | 1.2200 | 1.5594 | Cross-sex paternal half-sibling | 0.2054 | 0.0418 | 8.9*10^-7^ | 1.2280 | 1.1314 | 1.3329 |
| With **ADHD** | | | | | | | | | | | | | | | | | | | | |
|  |  |  |  |  |  |  |  |  |  |  |  |  |  | Mother-son | 0.9010 | 0.0155 | <2.2*10^-308^ | 2.4621 | 2.3883 | 2.5382 |
| Father-son | 0.8067 | 0.0161 | <2.2*10^-308^ | 2.2405 | 2.1709 | 2.3124 | Mother-daughter | 0.9150 | 0.0171 | <2.2*10^-308^ | 2.4969 | 2.4146 | 2.5819 | Father-daughter | 0.7505 | 0.0182 | <2.2*10^-308^ | 2.1181 | 2.0437 | 2.1952 |
| Male full sibling | 0.7179 | 0.0220 | 1.4*10^-233^ | 2.0502 | 1.9637 | 2.1405 | Female full sibling | 0.8450 | 0.0233 | 3.1*10^-287^ | 2.3279 | 2.2239 | 2.4368 | Cross-sex full sibling | 0.7396 | 0.0157 | <2.2*10^-308^ | 2.0951 | 2.0317 | 2.1605 |
| Male maternal half-sibling | 0.4130 | 0.0322 | 9.5*10^-38^ | 1.5113 | 1.4190 | 1.6096 | Female maternal half-sibling | 0.4421 | 0.0336 | 1.8*10^-39^ | 1.556 | 1.4568 | 1.6621 | Cross-sex maternal half-sibling | 0.3747 | 0.0233 | 2*10^-58^ | 1.4546 | 1.3898 | 1.5224 |
| Male paternal half-sibling | 0.3953 | 0.0323 | 2.2*10^-34^ | 1.4849 | 1.3937 | 1.5820 | Female paternal half-sibling | 0.4127 | 0.0342 | 1.6*10^-33^ | 1.5110 | 1.4130 | 1.6158 | Cross-sex paternal half-sibling | 0.3498 | 0.0241 | 1.2*10^-47^ | 1.4189 | 1.3533 | 1.4875 |

## **Table S11: Sensitivity analysis on familial risks of suicide attempt and self-harm among extended populations**

| **Suicide attempt** | | | | | | |
| --- | --- | --- | --- | --- | --- | --- |
|  | **Generalised Estimating Equations** | | | | | |
| **Relative types** | **Coefficient** | **SE** | **p-value** | **OR** | **95% CI of OR** | |
| Mother-offspring | 1.1853 | 0.0119 | <2.2*10^-308^ | 3.2716 | 3.1960 | 3.3490 |
| Father-offspring | 1.0158 | 0.0127 | <2.2*10^-308^ | 2.7615 | 2.6935 | 2.8312 |
| Full sibling | 1.1658 | 0.0149 | <2.2*10^-308^ | 3.2083 | 3.1161 | 3.3033 |
| Maternal half-sibling | 0.5861 | 0.0242 | 1,00*10^-129^ | 1.7970 | 1.7138 | 1.8843 |
| Paternal half-sibling | 0.4509 | 0.0247 | 1.3*10^-74^ | 1.5697 | 1.4956 | 1.6474 |

| **Self-harm** | | | | | | |
| --- | --- | --- | --- | --- | --- | --- |
|  | **Generalised Estimating Equations** | | | | | |
| **Relatedness** | **Coefficient** | **SE** | **p-value** | **OR** | **95% CI of OR** | |
| Mother-offspring | 1.0800 | 0.0100 | <2.2*10^-308^ | 2.9448 | 2.8874 | 3.0033 |
| Father-offspring | 0.9863 | 0.0103 | <2.2*10^-308^ | 2.6812 | 2.6275 | 2.7360 |
| Full sibling | 1.0838 | 0.0116 | <2.2*10^-308^ | 2.9559 | 2.8892 | 3.0241 |
| Maternal half-sibling | 0.5652 | 0.0204 | 4.2*10^-169^ | 1.7598 | 1.6909 | 1.8316 |
| Paternal half-sibling | 0.4114 | 0.0207 | 3.4*10^-88^ | 1.5089 | 1.4490 | 1.5713 |

*Note: Estimates for all sexes and included immigrants*

## **Table S12: Familial aggregation by relative’s age at first attempt and by sex**

| **Relative types** | **Age of onset in relative** | **Suicide attempt** | | | | | | **Self-harm** | | | | | | |
| --- | --- | --- | --- | --- | --- | --- | --- | --- | --- | --- | --- | --- | --- | --- |
|  |  | **Coefficient** | **SE** | **p-value** | **OR#** | **95% CI of OR** | | **Coefficient** | **SE** | | **p-value** | **OR#** | **95% CI of OR** | |
| **Males** |  |  | | | | | | | |  | | | | |
| Father-son | 10-18yr | 1.09 | 0.08 | 2.6*10^-47^ | 2.96 | 2.56 | 3.43 | 1.27 | 0.04 | | 3.7*10^-181^ | 3.55 | 3.26 | 3.87 |
|  | 19-25yr | 1.34 | 0.05 | 8.6*10^-182^ | 3.81 | 3.48 | 4.18 | 1.01 | 0.04 | | 1.9*10^-143^ | 2.75 | 2.54 | 2.97 |
|  | >25yr | 1.07 | 0.02 | <2.2*10^-308^ | 2.90 | 2.79 | 3.02 | 1.08 | 0.01 | | <2.2*10^-308^ | 2.94 | 2.86 | 3.03 |
| Male full sibling | 10-18yr | 1.23 | 0.06 | 1.8*10^-106^ | 3.43 | 3.07 | 3.82 | 1.56 | 0.04 | | <2.2*10^-308^ | 4.76 | 4.42 | 5.12 |
|  | 19-25yr | 1.33 | 0.04 | 7.9*10^-216^ | 3.79 | 3.49 | 4.12 | 1.20 | 0.03 | | <2.2*10^-308^ | 3.33 | 3.13 | 3.54 |
|  | >25yr | 1.12 | 0.04 | 5.6*10^-185^ | 3.05 | 2.83 | 3.29 | 1.01 | 0.03 | | 1.4*10^-282^ | 2.75 | 2.60 | 2.90 |
| Male maternal half-sibling | 10-18yr | 0.65 | 0.08 | 6.9*10^-15^ | 1.91 | 1.63 | 2.25 | 0.80 | 0.06 | | 2.6*10^-36^ | 2.23 | 1.97 | 2.53 |
|  | 19-25yr | 0.56 | 0.07 | 1.0*10^-14^ | 1.76 | 1.52 | 2.03 | 0.56 | 0.06 | | 5.6*10^-23^ | 1.75 | 1.57 | 1.96 |
|  | >25yr | 0.59 | 0.06 | 4.7*10^-20^ | 1.80 | 1.58 | 2.04 | 0.50 | 0.05 | | 3.6*10^-23^ | 1.65 | 1.49 | 1.82 |
| Male paternal half-sibling | 10-18yr | 0.56 | 0.09 | 6.4*10^-10^ | 1.75 | 1.46 | 2.09 | 0.61 | 0.07 | | 6.3*10^-18^ | 1.83 | 1.60 | 2.10 |
|  | 19-25yr | 0.49 | 0.07 | 5.3*10^-12^ | 1.64 | 1.42 | 1.88 | 0.47 | 0.06 | | 2.6*10^-14^ | 1.59 | 1.41 | 1.80 |
|  | >25yr | 0.46 | 0.06 | 7.4*10^-14^ | 1.58 | 1.40 | 1.78 | 0.37 | 0.05 | | 1.5*10^-13^ | 1.44 | 1.31 | 1.59 |
| **Females** |  |  | | | | | | | |  | | | | |
| Mother-daughter | 10-18yr | 1.30 | 0.04 | 2.7*10^-268^ | 3.69 | 3.43 | 3.97 | 1.22 | 0.03 | | <2.2*10^-308^ | 3.39 | 3.19 | 3.61 |
|  | 19-25yr | 1.28 | 0.04 | 2.4*10^-271^ | 3.58 | 3.34 | 3.85 | 1.10 | 0.03 | | 1.7*10^-238^ | 3.01 | 2.82 | 3.22 |
|  | >25yr | 1.22 | 0.02 | <2.2*10^-308^ | 3.40 | 3.28 | 3.53 | 1.12 | 0.02 | | <2.2*10^-308^ | 3.06 | 2.97 | 3.15 |
| Female full sibling | 10-18yr | 1.43 | 0.04 | <2.2*10^-308^ | 4.16 | 3.88 | 4.46 | 1.38 | 0.03 | | <2.2*10^-308^ | 3.96 | 3.73 | 4.20 |
|  | 19-25yr | 1.29 | 0.04 | 1.7*10^-251^ | 3.65 | 3.38 | 3.93 | 1.15 | 0.03 | | 2.1*10^-255^ | 3.15 | 2.95 | 3.37 |
|  | >25yr | 1.16 | 0.04 | 7.9*10^-189^ | 3.19 | 2.95 | 3.44 | 0.99 | 0.03 | | 4.2*10^-191^ | 2.68 | 2.51 | 2.87 |
| Female maternal half-sibling | 10-18yr | 0.69 | 0.06 | 3.3*10^-36^ | 2.00 | 1.79 | 2.23 | 0.70 | 0.05 | | 2.1*10^-44^ | 2.01 | 1.82 | 2.22 |
|  | 19-25yr | 0.58 | 0.06 | 1.5*10^-20^ | 1.78 | 1.58 | 2.01 | 0.58 | 0.06 | | 1.2*10^-23^ | 1.78 | 1.59 | 1.99 |
|  | >25yr | 0.60 | 0.06 | 1.6*10^-22^ | 1.82 | 1.61 | 2.05 | 0.54 | 0.05 | | 1.1*10^-23^ | 1.72 | 1.55 | 1.91 |
| Female paternal half-sibling | 10-18yr | 0.54 | 0.06 | 2.7*10^-20^ | 1.71 | 1.53 | 1.92 | 0.55 | 0.05 | | 1.1*10^-26^ | 1.73 | 1.57 | 1.92 |
|  | 19-25yr | 0.58 | 0.06 | 6.3*10^-20^ | 1.79 | 1.58 | 2.02 | 0.53 | 0.06 | | 7.4*10^-21^ | 1.70 | 1.52 | 1.89 |
|  | >25yr | 0.38 | 0.06 | 1.4*10^-09^ | 1.46 | 1.29 | 1.65 | 0.35 | 0.05 | | 4.3*10^-11^ | 1.42 | 1.28 | 1.57 |
| **Cross-sex** |  |  | | | | | | | |  | | | | |
| Mother-son | 10-18yr | 1.17 | 0.05 | 4.4*10^-114^ | 3.22 | 2.91 | 3.56 | 1.11 | 0.04 | | 7.5*10^-204^ | 3.04 | 2.83 | 3.26 |
|  | 19-25yr | 1.22 | 0.04 | 5.1*10^-206^ | 3.38 | 3.12 | 3.65 | 0.95 | 0.03 | | 8.0*10^-174^ | 2.59 | 2.42 | 2.77 |
|  | >25yr | 1.15 | 0.02 | <2.2*10^-308^ | 3.16 | 3.04 | 3.29 | 1.09 | 0.02 | | <2.2*10^-308^ | 2.98 | 2.89 | 3.08 |
| Father-daughter | 10-18yr | 1.07 | 0.06 | 3.9*10^-82^ | 2.92 | 2.62 | 3.26 | 1.12 | 0.04 | | 3.4*10^-160^ | 3.08 | 2.84 | 3.34 |
|  | 19-25yr | 1.15 | 0.05 | 1.4*10^-131^ | 3.17 | 2.89 | 3.48 | 0.95 | 0.04 | | 3.8*10^-110^ | 2.59 | 2.38 | 2.81 |
|  | >25yr | 0.94 | 0.02 | <2.2*10^-308^ | 2.57 | 2.47 | 2.67 | 0.91 | 0.02 | | <2.2*10^-308^ | 2.48 | 2.40 | 2.55 |
| Cross-sex full sibling | 10-18yr | 1.12 | 0.03 | 2.1*10^-261^ | 3.06 | 2.87 | 3.26 | 1.26 | 0.02 | | <2.2*10^-308^ | 3.52 | 3.35 | 3.69 |
|  | 19-25yr | 1.07 | 0.03 | 1.9*10^-286^ | 2.91 | 2.74 | 3.08 | 0.93 | 0.02 | | <2.2*10^-308^ | 2.53 | 2.42 | 2.65 |
|  | >25yr | 1.02 | 0.03 | 9.2*10^-295^ | 2.76 | 2.61 | 2.91 | 0.87 | 0.02 | | <2.2*10^-308^ | 2.39 | 2.29 | 2.50 |
| Cross-sex maternal half-sibling | 10-18yr | 0.51 | 0.05 | 2.9*10^-24^ | 1.66 | 1.51 | 1.83 | 0.59 | 0.04 | | 3.8*10^-49^ | 1.81 | 1.67 | 1.96 |
|  | 19-25yr | 0.59 | 0.05 | 1.0*10^-35^ | 1.80 | 1.64 | 1.98 | 0.55 | 0.04 | | 1.2*10^-43^ | 1.74 | 1.61 | 1.88 |
|  | >25yr | 0.52 | 0.04 | 5.2*10^-32^ | 1.69 | 1.55 | 1.84 | 0.45 | 0.04 | | 2.5*10^-34^ | 1.57 | 1.46 | 1.69 |
| Cross-sex paternal half-sibling | 10-18yr | 0.33 | 0.05 | 7.6*10^-10^ | 1.38 | 1.25 | 1.54 | 0.37 | 0.04 | | 3.7*10^-17^ | 1.44 | 1.33 | 1.57 |
|  | 19-25yr | 0.37 | 0.05 | 1.6*10^-12^ | 1.44 | 1.30 | 1.59 | 0.36 | 0.04 | | 9.8*10^-17^ | 1.43 | 1.32 | 1.56 |
|  | >25yr | 0.46 | 0.05 | 4.7*10^-24^ | 1.58 | 1.44 | 1.72 | 0.35 | 0.04 | | 2.5*10^-21^ | 1.42 | 1.32 | 1.53 |

## **Table S13: Intra-class correlations**

| **Sex groups** | **Type of sibling** | **Suicide attempt** | | **Self-harm** | |
| --- | --- | --- | --- | --- | --- |
|  |  | **Tetrachoric correlation** | **Standard error** | **Tetrachoric correlation** | **Standard error** |
| Both sexes | Full sibling | 0.2457 | 0.0034 | 0.2496 | 0.0028 |
|  | Maternal half-sibling | 0.1363 | 0.0061 | 0.1415 | 0.0054 |
| Male | Full sibling | 0.2531 | 0.0071 | 0.2761 | 0.0053 |
|  | Maternal half-sibling | 0.1313 | 0.0131 | 0.1426 | 0.0110 |
| Female | Full sibling | 0.2832 | 0.0062 | 0.2704 | 0.0055 |
|  | Maternal half-sibling | 0.1523 | 0.0116 | 0.1587 | 0.0107 |

## **Table S14: Heritability**

| **Suicide attempt** | | | | | | | | | |
| --- | --- | --- | --- | --- | --- | --- | --- | --- | --- |
|  | **ACE** | | | | **AE** | | | |  |
| **Component** | **Estimate** | **SE** | **95% CI** |  | **Estimate** | **SE** | **95% CI** |  | **Model fit^a^** |
| **Both sexes** |  |  |  |  |  |  |  |  |  |
| A | **0.4190** | **0.0318** | **0.3596** | **0.4838** | 0.4953 | 0.0073 | 0.4802 | 0.5096 | ACE 𝝌^2^=355.9637  AE 𝝌^2^=363.691  p-value=5.44*10^-3^ |
| C | **0.0357** | **0.0145** | **0.0067** | **0.0626** |  |  |  |  |  |
| E | **0.5453** | **0.0181** | **0.5099** | **0.5790** | 0.5047 | 0.0073 | 0.4904 | 0.5198 |  |
| **Male** |  |  |  |  |  |  |  |  |  |
| A | **0.4510** | **0.0527** | **0.3232** | **0.5249** | 0.500 | 0.0154 | 0.4712 | 0.5323 | ACE 𝝌^2^=72.9117  AE 𝝌^2^=73.6133  p-value=4.02*10^-1^ |
| C | **0.0230** | **0.0231** | **0.0001** | **0.0815** |  |  |  |  |  |
| E | **0.5260** | **0.0313** | **0.4736** | **0.5978** | 0.500 | 0.0154 | 0.4677 | 0.5288 |  |
| **Female** |  |  |  |  |  |  |  |  |  |
| A | **0.5144** | **0.0513** | **0.4012** | **0.5864** | 0.5704 | 0.0144 | 0.5401 | 0.5976 | ACE 𝝌^2^=109.5043  AE 𝝌^2^=110.6971  p-value=2.75*10^-1^ |
| C | **0.0263** | **0.0225** | **0.0001** | **0.0762** |  |  |  |  |  |
| E | **0.4593** | **0.0303** | **0.4129** | **0.5238** | 0.4296 | 0.0144 | 0.4024 | 0.4599 |  |
| Compare heritability (A) between female and male: Difference = 0.0634; SE difference 0.0747 (extracted from bootstrap resampling analysis); P-value^b^ = 0.3963 | | | | | | | | | |
| **Self-harm** | | | | | | | | | |
|  | **ACE** | | | | **AE** | | | |  |
| **Component** | **Estimate** | **SE** | **95% CI** | | **Estimate** | **SE** | **95% CI** | | **Model fit^a^** |
| **Both sexes** |  |  |  |  |  |  |  |  |  |
| A | **0.4226** | **0.0282** | **0.3698** | **0.4785** | 0.5043 | 0.0065 | 0.4917 | 0.5180 | ACE 𝝌^2^=496.3629  AE 𝝌^2^=508.1392  p-value=6.00*10^-4^ |
| C | **0.0386** | **0.0127** | **0.0140** | **0.0630** |  |  |  |  |  |
| E | **0.5389** | **0.0161** | **0.5081** | **0.5692** | 0.4957 | 0.0065 | 0.482 | 0.5083 |  |
| **Male** |  |  |  |  |  |  |  |  |  |
| A | **0.5231** | **0.0424** | **0.4173** | **0.5710** | 0.5546 | 0.0117 | 0.5309 | 0.5772 | ACE 𝝌^2^=104.3607  AE 𝝌^2^=104.7929  p-value=5.11*10^-1^ |
| C | **0.0150** | **0.0193** | **0.0001** | **0.0646** |  |  |  |  |  |
| E | **0.4619** | **0.0245** | **0.4278** | **0.5224** | 0.4454 | 0.0117 | 0.4228 | 0.4691 |  |
| **Female** |  |  |  |  |  |  |  |  |  |
| A | **0.4425** | **0.0526** | **0.3432** | **0.5420** | 0.5472 | 0.0124 | 0.5220 | 0.5708 | ACE 𝝌^2^=117.753  AE 𝝌^2^=122.7387  p-value=2.56*10^-2^ |
| C | **0.0493** | **0.024** | **0.0003** | **0.0942** |  |  |  |  |  |
| E | **0.5082** | **0.0299** | **0.4511** | **0.5658** | 0.4528 | 0.0124 | 0.4292 | 0.4780 |  |
| Compare heritability (A) between female and male: Difference = -0.0806; SE difference 0.0687 (extracted from bootstrap resampling analysis); P-value^b^ = 0.2407 | | | | | | | | | |

**SE and 95% CI estimated from Bootstrap resampling (among successful optimizations, i.e., Mx exit status code 0). ^a^difference in degree of freedom=1. p-value for testing whether the fit of the AE model is equal to that of the ACE model. p<0.05 rejects the equal fit and indicates AE model fit is worse than ACE. For consistency, estimates from ACE models (in* ***bolded text)*** *were presented in the main text*.

^b^Calculated as: 2*pnorm(-abs(diff/se_diff)), where diff is the difference in the whole-sample heritability estimate between female and male, se_diff is the standard deviation of 1000 differences in estimates of heritability from the bootstrap resampling analysis.

## **Table S15: Genetic correlations**

| **Suicide attempt** | | | | | | | | | |
| --- | --- | --- | --- | --- | --- | --- | --- | --- | --- |
|  | **ACE** | | | | **AE** | | | |  |
|  | ***r_g_*** | **SE** | **95% CI** | | ***r_g_*** | **SE** | **95% CI** | | **Model fit^b^** |
| Between male and female suicide attempt | **0.8513** | **0.0431** | **0.7953** | **0.9898** | 0.8480 | 0.0255 | 0.8010 | 0.8983 | ACE AIC: 3356386.3949  AE AIC: 3356381.7312  Difference in -2log likelihood: 1.3362  p-value^d^: 7.21*10^-1^ |
|  | p-value^a^ for testing *r_g_* **=** 1: <0.001 | | | | p-value^a^ for testing *r_g_* **=** 1: <0.001 | | | |  |
| ***r_g_* with** | ***r_g_*** | **SE** | **95% CI** | | ***r_g_*** | **SE** | **95% CI** | | **Model fit^c^** |
| Substance use disorder | **0.8545** | **0.0756** | **0.8338** | **0.9569** | 0.8815 | 0.0071 | 0.8676 | 0.8947 | ACE 𝝌^2^=1019.5176  AE 𝝌^2^=1019.5176  p-value^d^=3.08*10^-8^ |
| Major depressive disorder | **0.7669** | **0.0338** | **0.7226** | **0.8620** | 0.7742 | 0.0088 | 0.7575 | 0.7909 | ACE 𝝌^2^=899.9694  AE 𝝌^2^=911.4634  p-value^d^=9.33*10^-3^ |
| Anxiety | **0.6920** | **0.0471** | **0.6086** | **0.7919** | 0.6710 | 0.0117 | 0.6481 | 0.6929 | ACE 𝝌^2^=594.1906  AE 𝝌^2^=605.2162  p-value^d^=1.16*10^-2^ |
| OCD | **0.5087** | **0.0718** | **0.4119** | **0.6824** | 0.4321 | 0.0200 | 0.3951 | 0.4741 | ACE 𝝌^2^=453.4184  AE 𝝌^2^=463.1042  p-value^d^=2.14*10^-2^ |
| PTSD | **0.8008** | **0.0431** | **0.7747** | **0.9575** | 0.8365 | 0.0100 | 0.8168 | 0.8549 | ACE 𝝌^2^=614.9114  AE 𝝌^2^=640.4157  p-value^d^=1.21*10^-5^ |
| Eating disorder | **0.3665** | **0.0927** | **0.2508** | **0.6027** | 0.4459 | 0.0237 | 0.3989 | 0.4935 | ACE 𝝌^2^=520.5717  AE 𝝌^2^=529.7749  p-value^d^=2.67*10^-2^ |
| Schizophrenia | **0.4254** | **0.1206** | **0.3149** | **0.8189** | 0.4825 | 0.0283 | 0.4468 | 0.5194 | ACE 𝝌^2^=440.0048  AE 𝝌^2^=449.2474  p-value^d^=2.62*10^-2^ |
| Bipolar disorder | **0.6479** | **0.0650** | **0.5738** | **0.8250** | 0.6134 | 0.0136 | 0.5887 | 0.6430 | ACE 𝝌^2^=546.0805  AE 𝝌^2^=555.3302  p-value^d^=2.61*10^-2^ |
| Other psychotic disorders (excl. SCZ) | **0.6299** | **0.0764** | **0.5081** | **0.8067** | 0.6230 | 0.0157 | 0.5860 | 0.6473 | ACE 𝝌^2^=441.417  AE 𝝌^2^=453.3452  p-value^d^=7.63*10^-3^ |
| Autism spectrum disorder | **0.3893** | **0.0730** | **0.2584** | **0.5408** | 0.4192 | 0.0138 | 0.3935 | 0.4469 | ACE 𝝌^2^=649.1241  AE 𝝌^2^=679.7427  p-value^d^=1.02*10^-6^ |
| ADHD | **0.6245** | **0.0437** | **0.5454** | **0.7062** | 0.6361 | 0.0092 | 0.6209 | 0.6560 | ACE 𝝌^2^=895.3396  AE 𝝌^2^=923.4759  p-value^d^=3.40*10^-6^ |
| **Self-harm** | | | | | | | | | |
|  | **ACE** | | | | **AE** | | | |  |
|  | ***r_g_*** | **SE** | **95% CI** | | ***r_g_*** | **SE** | **95% CI** | | **Model fit^b^** |
| Between male and female self-harm | **0.8274** | **0.0562** | **0.7809** | **0.9980** | 0.8290 | 0.0204 | 0.7897 | 0.8712 | ACE AIC: 346458.4796  AE AIC: 4346456.8937  Difference in -2log likelihood: 4.4141  p-value^d^: 2.20*10^-1^ |
|  | p-value^a^ for testing *r_g_* **=** 1: <0.001 | | | | p-value^a^ for testing *r_g_* **=** 1: <0.001 | | | |  |
| ***r_g_* with** | ***r_g_*** | **SE** | **95% CI** | | ***r_g_*** | **SE** | **95% CI** | | **Model fit^c^** |
| Substance use disorder | **0.7269** | **0.0756** | **0.8338** | **0.9569** | 0.7640 | 0.0071 | 0.8676 | 0.8947 | ACE 𝝌^2^=1290.0425  AE 𝝌^2^=1318.775  p-value=2.55*10^-6^ |
| Major depressive disorder | **0.6058** | **0.0338** | **0.7226** | **0.8620** | 0.6300 | 0.0088 | 0.7575 | 0.7909 | ACE 𝝌^2^=693.87  AE 𝝌^2^=709.4038  p-value^d^=1.41*10^-3^ |
| Anxiety | **0.5483** | **0.0471** | **0.6086** | **0.7919** | 0.5483 | 0.0117 | 0.6481 | 0.6929 | ACE 𝝌^2^=721.2929  AE 𝝌^2^=735.1792  p-value^d^=3.06*10^-3^ |
| OCD | **0.3779** | **0.0718** | **0.4119** | **0.6824** | 0.3421 | 0.0200 | 0.3951 | 0.4741 | ACE 𝝌^2^=593.0331  AE 𝝌^2^=605.9688  p-value^d^=4.78*10^-3^ |
| PTSD | **0.6290** | **0.0431** | **0.7747** | **0.9575** | 0.6903 | 0.0100 | 0.8168 | 0.8549 | ACE 𝝌^2^=721.9193  AE 𝝌^2^=747.8095  p-value^d^=1.01*10^-5^ |
| Eating disorder | **0.2350** | **0.0927** | **0.2508** | **0.6027** | 0.3478 | 0.0237 | 0.3989 | 0.4935 | ACE 𝝌^2^=638.6882  AE 𝝌^2^=653.0184  p-value^d^=2.49*10^-3^ |
| Schizophrenia | **0.3309** | **0.1206** | **0.3149** | **0.8189** | 0.3833 | 0.0283 | 0.4468 | 0.5194 | ACE 𝝌^2^=570.8889  AE 𝝌^2^=583.5696  p-value^d^=5.38*10^-3^ |
| Bipolar disorder | **0.5297** | **0.0650** | **0.5738** | **0.8250** | 0.5049 | 0.0136 | 0.5887 | 0.6430 | ACE 𝝌^2^=687.7135  AE 𝝌^2^=700.6251  p-value^d^=4.83*10^-3^ |
| Other psychotic disorders (excl. SCZ) | **0.4947** | **0.0764** | **0.5081** | **0.8067** | 0.5126 | 0.0157 | 0.586 | 0.6473 | ACE 𝝌^2^=619.7394  AE 𝝌^2^=635.9256  p-value^d^=1.04*10^-3^ |
| Autism spectrum disorder | **0.2606** | **0.0730** | **0.2584** | **0.5408** | 0.3321 | 0.0138 | 0.3935 | 0.4469 | ACE 𝝌^2^=736.1114  AE 𝝌^2^=772.0262  p-value^d^=7.81*10^-8^ |
| ADHD | **0.5173** | **0.0437** | **0.5454** | **0.7062** | 0.5360 | 0.0092 | 0.6209 | 0.6560 | ACE 𝝌^2^=1035.6129  AE 𝝌^2^=1035.6129  p-value^d^=1.14*10^-6^ |

*^a^Empirical p-value for testing whether the r_g_ is <1 based on Bootstrap distribution; calculated as the proportion of (1-r_g_) ≤ 0 out of all valid Bootstrap replicates (Mx exit status code 0).*

*^b^difference in degree of freedom=3.*

*^c^difference in degree of freedom=3.*

^d^*P-value for testing whether the fit of the AE model is equal to that of the ACE model. P-value<0.05 rejects the equal fit and indicates AE model fit is worse than ACE. For consistency, estimates from ACE models (in* ***bolded text)*** *were presented in the main text*.

## **Table S16: Sex-specific genetic correlations**

| **Suicide attempt** | | | | | | | | | | | |
| --- | --- | --- | --- | --- | --- | --- | --- | --- | --- | --- | --- |
|  | **Male** | | | | **Female** | | | | **Compare female and male** | | |
| ***r_g_* with** | ***r_g_*** | **SE** | **95% CI** |  | ***r_g_*** | **SE** | **95% CI** |  | **Diff.** | **SE** | **P-value**^a^ |
| Substance use disorder | 0.8429 | 0.0263 | 0.8037 | 0.9104 | 0.8499 | 0.0318 | 0.8049 | 0.9372 | -0.0070 | 0.0412 | 0.8650 |
| Major depressive disorder | 0.7426 | 0.0363 | 0.6874 | 0.8364 | 0.7625 | 0.0698 | 0.6983 | 0.9597 | -0.0199 | 0.0781 | 0.7990 |
| Anxiety | 0.6198 | 0.0716 | 0.5259 | 0.811 | 0.6150 | 0.0617 | 0.5440 | 0.8046 | 0.0048 | 0.0953 | 0.9598 |
| OCD | 0.3290 | 0.0880 | 0.1883 | 0.5352 | 0.6660 | 0.1726 | 0.4070 | 0.9990 | -0.3370 | 0.1937 | 0.0819 |
| PTSD | 0.8394 | 0.0457 | 0.7486 | 0.9443 | 0.7945 | 0.0517 | 0.7431 | 0.9587 | 0.0449 | 0.0688 | 0.5138 |
| Eating disorder | 0.4219 | 0.1866 | 0.1432 | 0.9907 | 0.3306 | 0.1144 | 0.1308 | 0.5886 | 0.0913 | 0.2199 | 0.6780 |
| Schizophrenia | 0.5237 | 0.1991 | 0.2540 | 0.9990 | 0.4046 | 0.1205 | 0.2174 | 0.7208 | 0.1191 | 0.2330 | 0.6092 |
| Bipolar disorder | 0.4983 | 0.1184 | 0.3506 | 0.8518 | 0.5961 | 0.0441 | 0.5085 | 0.6753 | -0.0978 | 0.1251 | 0.4345 |
| Other psychotic disorders (excl. SCZ) | 0.6318 | 0.1199 | 0.4886 | 0.9925 | 0.5044 | 0.0934 | 0.3803 | 0.7577 | 0.1274 | 0.1542 | 0.4086 |
| Autism spectrum disorder | 0.4754 | 0.1326 | 0.2510 | 0.7851 | 0.3760 | 0.1161 | 0.1995 | 0.6551 | 0.0994 | 0.1746 | 0.5692 |
| ADHD | 0.6010 | 0.0431 | 0.5331 | 0.7092 | 0.5528 | 0.0544 | 0.4836 | 0.6922 | 0.0482 | 0.0691 | 0.4857 |
| **Self-harm** | | | | | | | | | | | |
|  | **Male** | | | | **Female** | | | | **Compare female and male** | | |
| ***r_g_* with** | ***r_g_*** | **SE** | **95% CI** | | ***r_g_*** | **SE** | **95% CI** | | **Diff.** | **SE** | **P-value**^a^ |
| Substance use disorder | 0.6276 | 0.0354 | 0.5690 | 0.7091 | 0.7544 | 0.0358 | 0.7010 | 0.8403 | -0.1268 | 0.0504 | 0.0118 |
| Major depressive disorder | 0.5119 | 0.0380 | 0.4375 | 0.5760 | 0.6793 | 0.0519 | 0.6009 | 0.7978 | -0.1674 | 0.0641 | 0.0090 |
| Anxiety | 0.4796 | 0.0770 | 0.3568 | 0.6658 | 0.5307 | 0.0705 | 0.4415 | 0.7154 | -0.0511 | 0.1064 | 0.6309 |
| OCD | 0.2067 | 0.0726 | 0.0980 | 0.4036 | 0.5750 | 0.1802 | 0.3101 | 0.9990 | -0.3683 | 0.1961 | 0.0604 |
| PTSD | 0.6004 | 0.0447 | 0.5312 | 0.7098 | 0.6845 | 0.0504 | 0.6322 | 0.8426 | -0.0841 | 0.0680 | 0.2162 |
| Eating disorder | 0.2465 | 0.1757 | 0.0128 | 0.7298 | 0.1897 | 0.1249 | -0.0489 | 0.4335 | 0.0568 | 0.2170 | 0.7935 |
| Schizophrenia | 0.4407 | 0.2014 | 0.1574 | 0.999 | 0.3566 | 0.1539 | 0.1414 | 0.7743 | 0.0841 | 0.2511 | 0.7377 |
| Bipolar disorder | 0.3534 | 0.0969 | 0.2436 | 0.6437 | 0.5150 | 0.0625 | 0.4123 | 0.6506 | -0.1616 | 0.1150 | 0.1600 |
| Other psychotic disorders (excl. SCZ) | 0.4123 | 0.0986 | 0.2980 | 0.6929 | 0.4366 | 0.1153 | 0.2913 | 0.7504 | -0.0243 | 0.1509 | 0.8720 |
| Autism spectrum disorder | 0.2933 | 0.1098 | 0.0856 | 0.5267 | 0.2949 | 0.1257 | 0.0966 | 0.5809 | -0.0016 | 0.1645 | 0.9922 |
| ADHD | 0.4175 | 0.0380 | 0.3538 | 0.5016 | 0.5013 | 0.0671 | 0.4051 | 0.6670 | -0.0838 | 0.0775 | 0.2797 |

*Note: Results from ACE models*

^a^Calculated as: 2*pnorm(-abs(diff/se_diff)), where diff is the difference in the whole-sample heritability estimate between female and male, se_diff is the standard deviation of 1000 differences in estimates of heritability from the bootstrap resampling analysis.

# **SUPPLEMENTARY FIGURES**

Individuals born in Sweden

during 1963-1998

(N=3,859,963)

Excluded

Death before age 10 (N=38,903)

First emigration before age 10 (N=124,440)

Adopted children (N=29,271)

Missing parent ID (N=29,344)

Excluded pairs where the relative died; migrated before age 10; did not reach 10 years old by 31-12-2019

Included index individuals

(N=3,058,374)

Parent-offspring (4,024,733 pairs)

Full-siblings (3,338,793 pairs)

Maternal half-siblings (592,797 pairs)

Paternal half-siblings (839,074 pairs)

Identified Sweden-born relatives

## **Figure S1: Sample size**

*After removing individuals who died and/or migrated before age 10, adopted individuals and those without information of both parents, we included 3,058,374 index individuals. We used Multi-Generation Register data to identify relatives who were born in Sweden. Relatives could be born outside the identified birth cohort period (1963-1998).*

***For the aggregation/coaggregation analyses****, we constructed a dataset separately for each type of relative where each pair appeared twice if both proband and relative were born within 1963-1998, with the role of proband and relative switched between the two individuals; and appeared once if the relative was born outside the 1963-1998 period. This resulted in:*

- ***3,653,013 mother-offspring pairs***
- ***3,477,548 father-offspring pairs***
- ***4,992,249 full sibling pairs***
- ***908,740 maternal half-sibling pairs***
- ***1,164,125 paternal half-sibling pairs***

***For the estimation of heritability and genetic correlation****s using SEM, to ensure the quality of phenotype for both siblings, we included pairs where both siblings were born within the cohort 1963-1998. The final analyses included* ***2,143,644*** ***full sibling pairs*** *and* ***343,075 maternal half-sibling pairs****.*

# **References**

1. Kendler KS, Ohlsson H, Mościcki EK, et al. Genetic liability to suicide attempt, suicide death, and psychiatric and substance use disorders on the risk for suicide attempt and suicide death: a Swedish national study. *Psychol Med* 2023;53(4):1639-48. doi: 10.1017/s0033291721003354 [published Online First: 2023/04/04]

2. Edwards AC, Ohlsson H, Mościcki E, et al. On the Genetic and Environmental Relationship Between Suicide Attempt and Death by Suicide. *Am J Psychiatry* 2021;178(11):1060-69. doi: 10.1176/appi.ajp.2020.20121705 [published Online First: 2021/07/15]

3. Kendler KS, Ohlsson H, Sundquist J, et al. The Sources of Parent-Child Transmission of Risk for Suicide Attempt and Deaths by Suicide in Swedish National Samples. *Am J Psychiatry* 2020;177(10):928-35. doi: 10.1176/appi.ajp.2020.20010017 [published Online First: 2020/08/29]

4. Cho H, Guo G, Iritani BJ, et al. Genetic Contribution to Suicidal Behaviors and Associated Risk Factors among Adolescents in the U.S. *Prevention Science* 2006;7(3):303-11. doi: 10.1007/s11121-006-0042-5

5. Baldessarini RJ, Hennen J. Genetics of suicide: an overview. *Harv Rev Psychiatry* 2004;12(1):1-13. doi: 10.1080/10673220490425915 [published Online First: 2004/02/18]

6. Fu Q, Heath AC, Bucholz KK, et al. A twin study of genetic and environmental influences on suicidality in men. *Psychological Medicine* 2002;32(1):11-24. doi: 10.1017/S0033291701004846 [published Online First: 2002/02/05]

7. Glowinski AL, Bucholz KK, Nelson EC, et al. Suicide attempts in an adolescent female twin sample. *J Am Acad Child Adolesc Psychiatry* 2001;40(11):1300-7. doi: 10.1097/00004583-200111000-00010 [published Online First: 2001/11/09]

8. Statham DJ, Heath AC, Madden PAF, et al. Suicidal behaviour: an epidemiological and genetic study. *Psychological Medicine* 1998;28(4):839-55. doi: 10.1017/S0033291798006916 [published Online First: 1998/07/01]

9. Ludvigsson JF, Almqvist C, Bonamy AK, et al. Registers of the Swedish total population and their use in medical research. *Eur J Epidemiol* 2016;31(2):125-36. doi: 10.1007/s10654-016-0117-y [published Online First: 2016/01/16]

10. Cnattingius S, Källén K, Sandström A, et al. The Swedish medical birth register during five decades: documentation of the content and quality of the register. *Eur J Epidemiol* 2023;38(1):109-20. doi: 10.1007/s10654-022-00947-5 [published Online First: 2023/01/04]

11. Ludvigsson JF, Andersson E, Ekbom A, et al. External review and validation of the Swedish national inpatient register. *BMC Public Health* 2011;11:450. doi: 10.1186/1471-2458-11-450 [published Online First: 2011/06/11]

12. Ekbom A. The Swedish Multi-generation Register. *Methods Mol Biol* 2011;675:215-20. doi: 10.1007/978-1-59745-423-0_10 [published Online First: 2010/10/16]

13. Brooke HL, Talbäck M, Hörnblad J, et al. The Swedish cause of death register. *Eur J Epidemiol* 2017;32(9):765-73. doi: 10.1007/s10654-017-0316-1 [published Online First: 2017/10/07]
